# Effects of anti-diabetic medications on the teeth, oral soft and hard tissues, saliva, and implants: a scoping review

**DOI:** 10.1038/s41405-026-00431-2

**Published:** 2026-07-01

**Authors:** Arya Agarwal, Marwa Khalil, Aditi Chopra

**Affiliations:** 1 Department of Periodontology, Manipal College of Dental Sciences, Manipal Academy of Higher Education, Manipal, India; 2 Department of Oral Medicine, Periodontology, Oral Diagnosis and Oral Radiology, Faculty of Dentistry, Alexandria University, Alexandria, Egypt

**Keywords:** Gum disease, Dental pharmacology, Periodontitis

## Abstract

Diabetes mellitus (DM) is one of the world’s most common non-communicable diseases. DM is a group of metabolic disorders in which blood glucose levels increase due to reduced insulin production or action, resulting in hyperglycemia. Various anti-diabetic medications, including insulin, metformin, glimepiride, glipizide, and thiazolidinedione, are used to control hyperglycemia in diabetic patients. These anti-diabetic medications have been shown to affect the gingiva, bone, fibroblast cells, pulpal tissues, dental stem cells, saliva, teeth, and oral microbiome. Metformin (a biguanide), insulin, and oral sulfonylureas (glipizide and glimepiride) have regenerative and host-modulating effects. Metformin and insulin can promote the growth of osteoblasts (bone-forming cells) and periodontal ligament fibroblasts and enhance the differentiation of mesenchymal stem cells to repair the lost periodontal tissues. Metformin is used in both systemic and local forms as a gel or scaffolds along with periodontal therapy (scaling and root planing) and surgical procedures to promote repair of periodontal tissues, alveolar bone defects, furcation defects, and osseointegration of dental implants. Although the effect of metformin on oral tissues is well established, the role of other anti-diabetic drugs on the oral and periodontal tissues is not comprehensively discussed. Thus, this review aims to discuss the positive and negative effects of various anti-diabetic medications on the oral cavity.

## Introduction

DM is considered one of the most common non-communicable metabolic disoder globally. DM is categorized as a group of metabolic disorders where individuals have high glucose levels (hyperglycemia) in the blood [1]. This is attributed to either reduced or absent secretion of insulin from the pancreas, altered or defective insulin action, or insulin resistance, or both [1, 2]. There are various forms of DM, such as Type 1 or Type 2 DM, gestational DM, or maturity-onset DM in young or adolescent individuals [3]. Among these, Type 2 DM is the most prevalent form worldwide [4, 5]. It is noted that in the United States, approximately 23.4 million adults are diagnosed with DM annually. Among these, 90 to 95% are suffering from Type 2 DM, 81.6 million from prediabetes, and ≈18,000 people under age 20 years have Type 1 DM [6].

The burden of DM is expected to rise exponentially in the coming years. It is estimated that by 2045, around 783 million people will have DM, increasing the risk of mortality and morbidity [7]. With a marked rise in DM expected, it is crucial to prevent, diagnose, and manage it at an early stage. It is also important to understand the various complications associated with DM and how it can be prevented. Neuropathy, nephropathy, retinopathy, diabetic foot ulcers, skin infections, hearing impairment, and Alzheimer’s disease are some of the known complications of DM. Many oral and periodontal conditions, such as increased risk of caries, altered salivary functions, xerostomia, bacterial and fungal infections such as increased risk of candidial infection, periodontal inflammation (gingivitis and periodontitis), rate of bone loss, tooth mobility, multiple periodontal abscesses, and early tooth loss and edentulism [8–12].

Periodontitis and multiple periodontal abscesses are considered the 6th most common manifestations of DM. Increased oral and periodontal inflammation in DM patients is attributed to the increased formation and deposition of various glycation adducts, such as ‘advanced glycation end products’ (AGEs) in the oral and periodontal tissues, along with an increased free radical or reactive oxygen species production and alteration in the oral microbiome [12, 13]. Apart from decreasing inflammation and inducing microbial changes, chronic use of anti-diabetic medications such as metformin (MF), insulin, glimepiride, glipizide, and thiazolidinedione also has regenerative and host-modulating effects on the oral tissues and their functions [14].

**Fig. 1:**
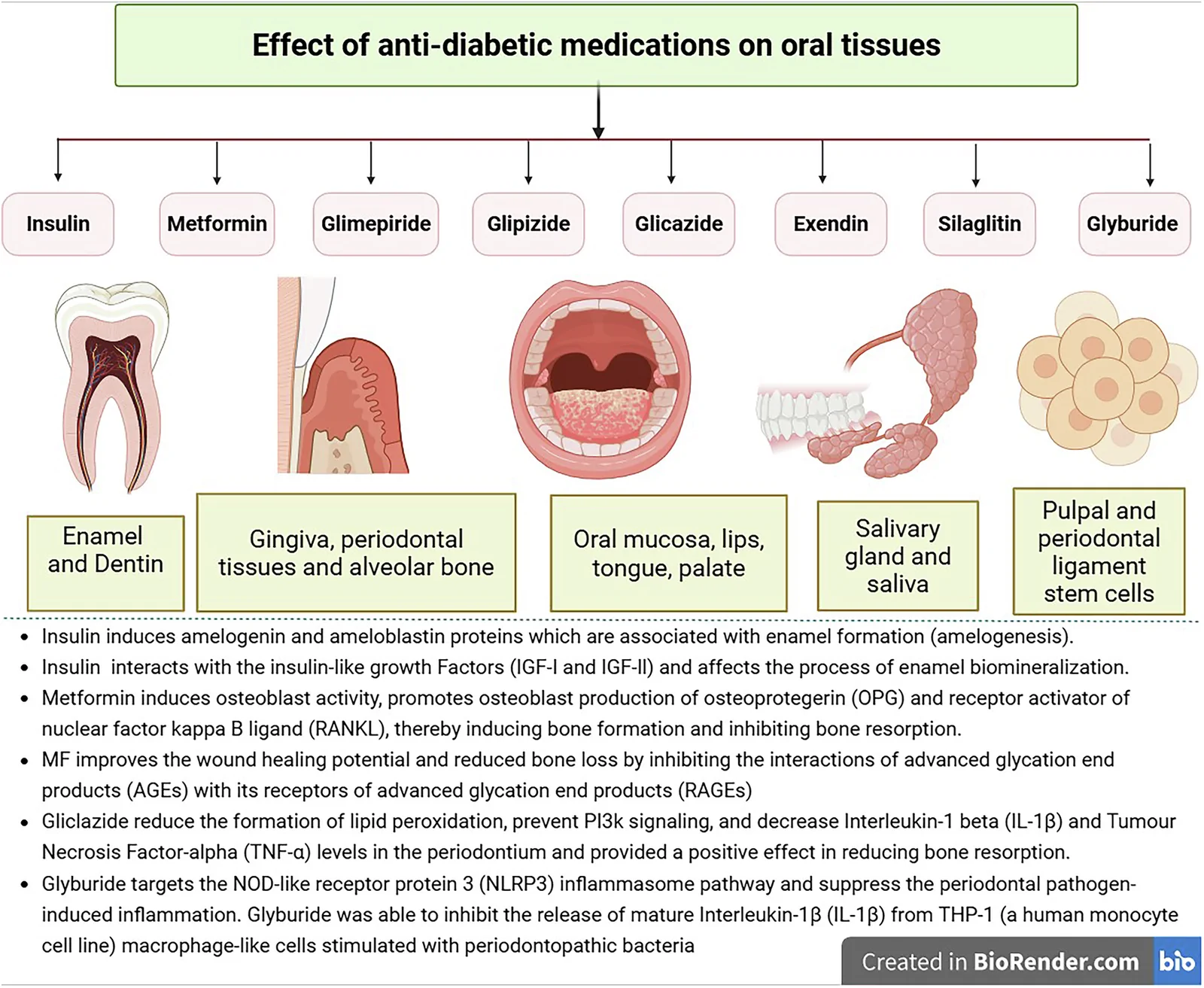
Effects of various anti-diabetic drugs on oral tissues.

**Fig. 2:**
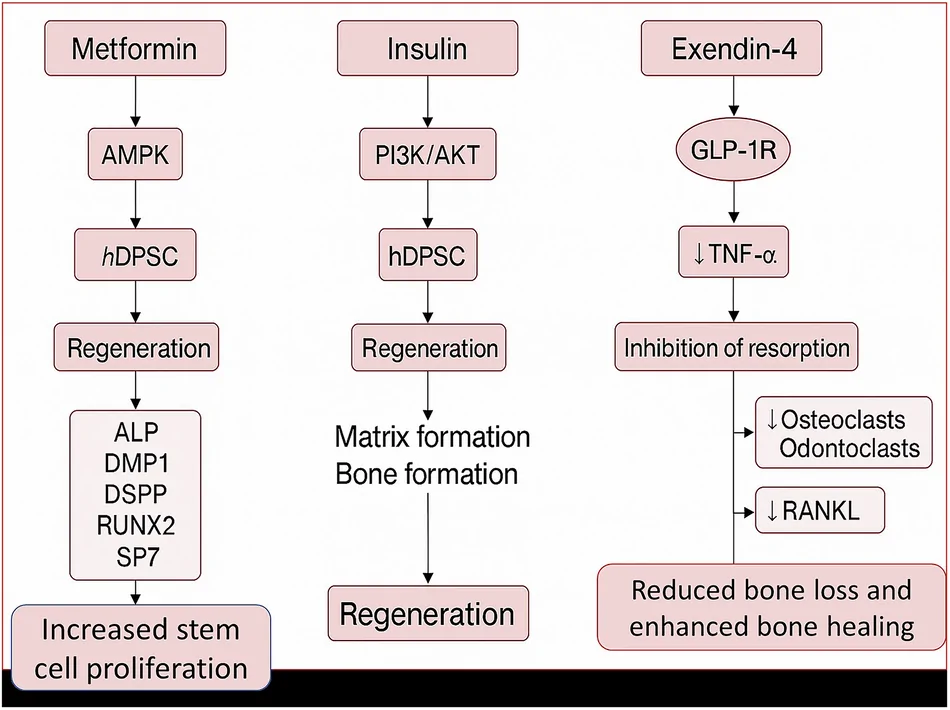
Effects of anti-diabetic drugs on teeth [enamel, dentin, pulp] and human dental pulp stem cells.

Studies have found that anti-diabetic medications have dual (positive and negative) effects on the oral and periodontal tissues, saliva, teeth, peri-implant tissue, osseointegration of the implant, and oral microbiome [14–20] [Fig. 1]. Anti-diabetic medications like MF and insulin have regenerative and host-modulating effects. MF and insulin can promote the growth of osteoblasts (bone-forming cells) and periodontal ligament fibroblasts and enhance the differentiation of mesenchymal stem cells to repair the lost periodontal tissues [Figs. 1 and 2]. MF in both systemic and local forms has been used as an adjunct to non-surgical periodontal therapy (scaling and root planing (SRP)) for controlling periodontal inflammation and promoting regneration and repair of lost periodontal tissues [17]. Given that Type 2 DM accounts for 90% of DM cases globally and chronic use of anti-diabetic medications is common [14], understanding the precise dual effects: positive, like MF’s regenerative action [11], and negative, like the potential for certain thiazolidinediones to reduce bone mineral density [19, 20], is critically important for oral health specialists. This knowledge is essential for guiding clinical decisions related to periodontal therapy, bone grafting, and dental implant osseointegration. Though the effects of DM on the periodontium and oral tissues are well established, the effects of anti-diabetic drugs on the oral cavity, which includes teeth, gingiva, periodontium, salivary glands, and peri-implant tissues, are not comprehensively discussed. This review aims to highlight and discuss the both the positive and negative effects of various anti-diabetic medications on the oral cavity.

## Search strategy, eligibility criteria, and selection of sources of evidence

The review was conducted following PRISMA-Scoping review guidelines. Five search engines [PubMed, Web of Science, Scopus, EMBASE, and Cochrane database] were searched using the following keywords: “anti-diabetic medications” OR “anti-diabetic drug* OR “oral hypoglycemic*” OR Metformin OR insulin OR “oral sulfonylureas” OR glimepiride OR glipizide OR glyburide OR Gliclazide AND “Oral cavity” OR periodontium OR “periodontal tissues” OR “gingival fibroblast” OR “oral health” OR saliva OR teeth OR enamel OR dentin OR alveolar bone OR “periodontal ligament” OR “gingival tissues” OR gingiva OR Pulp* OR “peri-implant*” OR “dental implant*”.

The search was first conducted in September 2024 and updated in April 2025. All the articles were compiled in the Rayyan software, and duplicates were removed. After the removal of duplicates, the authors (AA, AC) independently performed the title and abstract screening for all the articles, and in case of any discrepancy, a discussion with the third reviewer was made. The studies were screened using the following eligibility criteria: All in-vitro, in-vivo, animal studies, observational (cross-sectional, prospective, retrospective studies), and analytical studies were included. Any study reporting the effect of any anti-diabetic medication used in any form, mode of administration/ delivery, dose and reporting the effect on any of the following oral tissues: gingiva; periodontium (periodontal ligament, cementum, and alveolar bone); oral mucosa; buccal mucosa; tongue, palate; lips; salivary gland; teeth (enamel, dentin, roots, and pulp) were included. Articles where anti-diabetic drugs are used as an adjunct to non-surgical periodontal therapy (SRP) or surgical therapy, but no effect of the drug on the teeth, pulp, mesenchymal stem cell, palate, mucosa, gingiva, periodontal ligament, cementum, alveolar bone, saliva or salivary glands, tongue, or lips” were mentioned were excluded (Supplementary file 1: List of excluded articles). Any articles whose full text could not be retrieved or was unavailable [online or in print] were excluded. All articles up to 2025 published in English were included.

## Data charting

The following data/outcomes variables were extracted:

1. Characteristics of the individual studies: bibliographic and study details, i.e., author details, aim and objectives of the study, type of study design; participants’ details, i.e., number (sample size), age, gender, animal model/type, nature, type, mode, dosage, and frequency of application of anti-diabetic medication; type of cells/tissues/organs affected.

2. Effect of anti-diabetic medications on the teeth, gingiva, periodontal tissues, alveolar bone, implants, saliva, and development of autoimmune conditions.

3. The patient-reported outcomes and the presence of any side effects were noted with any anti-diabetic medication on the oral tissues Table 1.

**Table 1:** Search Strategy for different databases for including articles for the title and abstract screening.

S/No	Database	Search string used	No of articles
1	PubMed	[anti-diabetic medications[Title/Abstract] OR “anti-diabetic drug*“[Title/Abstract] OR “oral hypoglycemic*“[Title/Abstract] OR Metformin[Title/Abstract] OR insulin[Title/Abstract] OR “oral sulfonylureas”[Title/Abstract] OR glimepiride[Title/Abstract] OR glipizide[Title/Abstract] OR glyburide[Title/Abstract] OR Gliclazide[Title/Abstract] AND [“Oral cavity”[Title/Abstract] OR periodontium[Title/Abstract] OR “periodontal tissues”[Title/Abstract] OR “gingival fibroblast”[Title/Abstract] OR “oral health”[Title/Abstract] OR saliva[Title/Abstract] OR teeth[Title/Abstract] OR enamel[Title/Abstract] OR Dentin[Title/Abstract] OR alveolar bone[Title/Abstract] OR periodontal ligament[Title/Abstract] OR gingival tissues[Title/Abstract] OR gingiva[Title/Abstract] OR Pulp*[Title/Abstract]	1711
2	Scopus	TITLE-ABS-KEY [“anti-diabetic medications” OR “anti-diabetic drug*“ OR “oral hypoglycemic*“ OR metformin OR insulin OR “oral sulfonylureas” OR glimepiride OR glipizide OR glyburide OR gliclazide] AND TITLE-ABS-KEY [“Oral cavity” OR periodontium OR “periodontal tissues” OR “gingival fibroblast” OR “oral health” OR saliva OR teeth OR enamel OR dentin OR “alveolar bone” OR “periodontal ligament” OR “gingival tissues” OR gingiva OR pulp* OR “peri-implant*“ OR “dental implant*“]	6717
3	Embase	[‘anti-diabetic medications’:ti,ab,kw OR ‘anti-diabetic drug*‘:ti,ab,kw OR ‘oral hypoglycemic*‘:ti,ab,kw OR metformin:ti,ab,kw OR insulin:ti,ab,kw OR ‘oral sulfonylureas’:ti,ab,kw OR glimepiride:ti,ab,kw OR glipizide:ti,ab,kw OR glyburide:ti,ab,kw OR gliclazide:ti,ab,kw] AND [‘oral cavity’:ti,ab,kw OR periodontium:ti,ab,kw OR ‘periodontal tissues’:ti,ab,kw OR ‘gingival fibroblast’:ti,ab,kw OR ‘oral health’:ti,ab,kw OR saliva:ti,ab,kw OR teeth:ti,ab,kw OR enamel:ti,ab,kw OR dentin:ti,ab,kw OR ‘alveolar bone’:ti,ab,kw OR ‘periodontal ligament’:ti,ab,kw OR ‘gingival tissues’:ti,ab,kw OR gingiva:ti,ab,kw OR pulp*:ti,ab,kw OR ‘peri-implant*‘:ti,ab,kw OR ‘dental implant*‘:ti,ab,kw]	2197
4	Web of Science	“anti-diabetic medications” OR “anti-diabetic drug*“ OR “oral hypoglycemic*“ OR metformin OR insulin OR “oral sulfonylureas” OR glimepiride OR glipizide OR glyburide OR gliclazide [Title] and “Oral cavity” OR periodontium OR “periodontal tissues” OR “gingival fibroblast” OR “oral health” OR saliva OR teeth OR enamel OR dentin OR “alveolar bone” OR “periodontal ligament” OR “gingival tissues” OR gingiva OR pulp* OR “peri-implant*“ OR “dental implant*“ [Title]	206
		**Total Search** **Removal of duplicates** **Final articles for Title-Abstract screening** **Full text screening** **Excluded** **Included**	**10891** **7830** **3061** **177** **131** **46**

The results from each study and the characteristics of the included studies are reported in Table 2, and a description of the factors is provided as a narrative synthesis below as follows:

**Table 2:** Characteristics of individual studies.

S. no	Author name	Country	Type of Study	Aim	Type of anti-diabetic medication	Sample Size and grouping	Methodology	Result and Conclusion
1.	Takahashi et al., 1998 [21]	Japan	Animal study	Induction of Amelogenin and Ameloblastin by Insulin	Insulin	Insulin [1000 ng/ml], IGF-I [100 ng/ml] or IGF-II [100 ng/ml]	Mandibular first molars from Swiss Webster mice were placed in the presence of insulin [1000 ng/ml], IGF-I [100 ng/ml], or IGF-II [100 ng/ml] for 6, 12, and 18 days. On the 18th day, the concentration of mRNAs for amelogenin, tuftelin, and ameloblastin was determined using PCR.	IGF-1 increased the yield of amelogenin by 5.7-fold and ameloblastin by 2.8-fold. Treatment with IGF-2 increased the yield of amelogenin and ameloblastin mRNA by 7.4-fold and 4.2-fold, respectively [*p* < 0.05].
2.	Willis et al., 1999 [59]	United Kingdom	In vivo	Candidal load and carriage of candidal species in insulin-treated diabetes mellitus patients with and without clinical signs of infection.	Insulin	Candidal species were recovered from 414 insulin-treated diabetes mellitus patients attending two hospital diabetic clinics, using an oral rinse technique.	183 patients completed oral clinical examination after being shown to carry candidal species in the oral cavity with the oral rinse technique. Where erythematous candidosis was apparent. Blood samples were assayed for glycosylated haemoglobin concentration. Fructosamine levels (nondiabetic range 1.8 ± 2.8 mmol/l) in the blood measured by the reduction of alkaline solutions of nitroblue tetrazolium. Urine samples were analysed for glucose, protein, and ketones. The identification of all candidal species was confirmed by the conventional methods of germ tube production in serum the production of chlamydospores on cornmeal agar, and by the pattern of assimilation of a variety of carbon and nitrogen sources	*C. dubliniensis* was found for the first time in this patient group. 40% patients with candidal species had no clinical signs of oral candidiasis. And if present, erythematous candidiasis was the most common. Oral candidiasis in insulin-treated diabetes mellitus patients is not the result of a single entity, but a combination of risk factors.
3.	Caldeira et al., 2005 [52]	Brazil	Animal Study	To determine the effects of prolonged insulin treatment on the morphology of the salivary glands in Non-obese diabetic (Nod mice).	Insulin	45 female mice were divided into five groups: nine positive diabetic Nod mice for 10 days (group 1), nine positive diabetic Nod mice for 20 days (group 2), nine diabetic Nod mice for 10 days (group 3), nine diabetic Nod mice for 20 days (group 4), and nine nondiabetic BALB/c mice (group 5).	Animals of groups 3 and 4 received 4–5 U of insulin daily, whereas animals of groups 1, 2, and 5 received the same dose of physiological saline simulating the experimental conditions. Samples of the salivary glands were analyzed by light, transmission, and scanning electron microscopies.	Intense alterations in diabetic animals characterized by nuclear and cytoplasmic atrophy, biomembrane disorganization, an increase in fibrillar components of the extracellular matrix, and the presence of inflammatory cells. Insulin treatment exerted positive effects on the recovery of the changes resulting from the diabetic state in both parotid and submandibular glands, but the pattern continued to be altered.
4.	Cortizo et al., 2006 [29]	Argentina	In-vitro	Evaluated the effects of MF on the growth and differentiation of osteoblast cells.	MF	Two osteoblast-like cells were treated with MF [25–500 μM] for 24 h	UMR106 rat osteosarcoma cells and MC3T3E1 mouse calvaria-derived cells were cultured with 5 mM β-glycerol-phosphate and 145 μM ascorbic acid to promote alkaline phosphatase formation. Cells on multi-well plates were treated with various doses of MF. For experiments on type-I collagen secretion and mineralization with MC3T3E1 osteoblasts, cells were cultured for 2 or 3 weeks, respectively, with or without different doses of MF. For alkaline phosphatase expression studies with MC3T3E1 osteoblasts, cells were cultured for 2 weeks in DMEM/FBS supplemented with β-glycerol-phosphate and ascorbic acid, with the medium changed every 2 days. Afterwards, they were serum-starved and incubated in DMEM with or without various doses of MF for an additional 24 h. Cell proliferation was measured using the crystal violet mitogenic bioassay. Osteoblastic differentiation was assessed via alkaline phosphatase activity and quantification of type-I collagen production. The regulation of ERK 1/2 activation by MF, along with eNOS and iNOS expression, was evaluated via Western blot. Localization of P-ERK and i/eNOS was checked by immunofluorescence.	MF led to a dose-dependent increase in cell proliferation. MF also promoted osteoblastic differentiation and increased type-I collagen production and stimulated alkaline phosphatase activity. In addition, MF markedly increased the formation of nodules of mineralization in 3-week MC3T3E1 cultures. MF induced activation and redistribution of phosphorylated extracellular signal-regulated kinase [P-ERK] in a transient manner, and dose-dependently stimulated the expression of endothelial and inducible nitric oxide synthases [e/iNOS]. These results show for the first time a direct osteogenic effect of MF on osteoblasts in culture, which could be mediated by activation/redistribution of ERK-1/2 and induction of e/iNOS.
5.	McCracken et al. (2005) [46].	USA	Animal study	Measure bone response to implants in uncontrolled and insulin-controlled diabetic rats.	Insulin	152 rats were divided into control, diabetic, and insulin groups. Rats received streptozotocin (65 mg/kg) to induce diabetes; animals in the insulin group also received a subcutaneous slow-release insulin implant.	Titanium alloy implants (1.5 ×8 mm) were placed in the proximal tibiae of animals. Implants were harvested at 2, 7, 14, and 24 days and examined histologically. Bone or bone-like tissue adjacent to implants was quantified as a percent.	Time and treatment were significant factors in predicting bone response to implants (*P* < 0.0001). Mean bone volume peaked at day 7 and decreased over time to day 24. Mean bone volume percent at 2, 7, 14, and 24 days (±SD) was 8.2 (±8), 22.9 (±8), 18.8 (±10), and 14.9 (±9), respectively. Mean total bone volume percent (adjusted for day) for control, diabetic, and insulin groups (±SD) was 12.4 (±9), 22.6 (±10), and 17 (±7), respectively. Bone volume adjacent to implants in diabetic rats was significantly greater than controls (*P* < 0.05). Diabetic animals treated with insulin were not statistically different from controls. Induction of diabetes with STZ is associated with increased bone response compared with controls.
6.	de Morais et al., 2009 [43]	Brazil	Animal study	To determine how DM and insulin therapy affect the bone density around osseointegrated dental implants.	Insulin	40 implants were placed in the tibiae of 40 adult rats. The animals were divided into four groups [*N* = 10 in each group]: control group without DM and insulin treatment sacrificed at 2 months; DM group without any treatment; DM group treated with insulin-treated group and sacrificed at 4 months; and DM group treated with saline.	Plasma glucose levels were monitored throughout the experiment. Film radiographs were taken at implant surgery, and bone density in the osseointegration regions around the implants was evaluated using quantitative Digital Subtraction Radiography (DSR) between baseline and final images.	The levels of glucose were higher at the end of 4 months for DM rats fed with saline alone. New bone was formed along the implant threads in all groups of animals; however, the bone formed was thinner in the group with saline than in the group with insulin. After 4 months, a reduced bone density around implants in diabetic rats compared with insulin-treated rats was noted. DM impaired bone density around the dental implant. The use of insulin could help to maintain bone density in DM.
7.	Bak et al., 2010 [35]	Korea	Animal study	Assess the effect of MF on alveolar bone loss	MF	Two groups: Rats with ligature receiving saline [*n* = 5] and Rats with ligature receiving MF [*n* = 5].	Periodontitis was induced by a ligature around the mandibular 1st molar of each rat. On the 10th day, the alveolar bone volume between the 1st and 2nd molars was determined via microcomputed tomography. The effect of MF on osteoblast, osteoclast, and adipocyte differentiation was assessed.	MFs exert a beneficial effect on alveolar bone in periodontitis by increasing osteoblast differentiation. MF augmented the mineralization of osteoblast precursor cell line derived from Mus musculus [mouse] calvaria [MC3T3-E1 cells] by approximately twofold over the non-treated cells. No effects on adipocyte differentiation and osteoclast formation induced by 1,25-dihydroxyvitamin D_3_, lipopolysaccharide, and PGE_2_ were noted.
8.	Han et al., 2012 [34]	China	In-vitro study	Insulin/poly (lactic-co-glycolic acid) (PLGA) microspheres incorporated in fibrin gel to develop a local drug delivery system for diabetic patients requiring implant treatment. G ability of human MG-63 cells under high glucose conditions. Fibrin gel loaded with insulin/PLGA microspheres shows potential for improving peri-implant-bone formation in diabetic patients.	Insulin	The human MG-63 cell suspensions were diluted to 10000 cells mL-1 in Minimum essential medium with Earle’s balanced salts (MEM/EBSS) MEM/EBSS containing 10% FBS and seeded onto 96-well plates to a final well volume of 200 μL (2000 cells/well). After culturing for 24 h, the cells were serum-starved overnight in serum-free MEM/EBSS. The medium was then exchanged, and cells treated with one of the following media: a MEM/EBSS containing NEAA, 5% FBS and 5.5 mmol L-1 glucose (normal glucose, NG); b. MEM/EBSS containing NEAA, 5% FBS and 25 mmol L-1 glucose (high glucose, HG); c. MEM/EBSS containing NEAA, 5% FBS, 25 mmol L -1 glucose and 100 nmol L-1 fresh insulin; d. MEM/EBSS containing NEAA, 5% FBS, 25 mmol L-1 glucose and 100 nmol L-1 insulin released from fibrin gel loaded with insulin/PLGA microspheres; and e. MEM/EBSS containing NEAA, 5% FBS, 25 mmol L-1 glucose and supernatant from fibrin gel loaded with placebo PLGA microspheres.	In vitro release of insulin from fibrin gel loaded with these microspheres was assessed, and sustained prolonged insulin release over 21 days. To assess the bioactivity of released insulin and determine whether slow release might improve impaired diabetic bone formation, 3-(4,5-dimethylthiazol-2-yl)-2,5-diphenyltetrazolium bromide (MTT), alkaline phosphatase (ALP) activity, mineralized nodule formation, and ELISA (enzyme-linked immunosorbent assay) assays were performed.	After treatment of cells with high levels of glucose and insulin, collagen type I was significantly increased (*P* < 0.001). Additionally, there was no difference in the secretion of collagen type I between the cells treated with released insulin or fresh insulin. The placebo group showed no change in collagen type I secretion under high glucose. The amount of collagen type I from the cells treated with supernatants containing released insulin was significantly higher than those of the cells treated with supernatants containing no insulin, i.e., the placebo group (*P* < 0.01). Fibrin gel loaded with insulin/PLGA microspheres shows potential for improving peri-implant-bone formation in diabetic patients.
9.	Wang et al., 2017 [20]	China	Animal Study	Investigated the influence of local infiltration of insulin at the implant-bone interface after implantation in diabetic rats.	Insulin	GK rats [8-week-old *N* = 20] with Type 2 DM, and Sprague-Dawley rats as controls [*n* = 10]. GK rats were divided into two groups: those with DM alone and those with DM given insulin [*n* = 10 in each group].	The insulin group was given controlled-release insulin at the implant-bone interface. At 2 and 6 weeks after implant placement, bone-implant contact and bony volume were checked in all rats.	Implant-bone contact, osteoid and osteogenic volume, and the amount of newly formed bone in the DM group were significantly lower than in the control [*p* < 0.05] and Insulin [*p* < 0.01] groups. Implant-bone contact in the insulin group was less than in the control group, but the amount of newly formed bone was higher. Implant-bone contact in the insulin group did not reach the level of the control, direct insulin infiltration could improve implant-bone contact.
10.	Silva Faria et al. [2012] [51]	Brazil	Animal study	Evaluate the treatment with MK0431 [Sitagliptin] in the salivary glands of diabetic mice	Sitagliptin	10 animals each in group I [NOD diabetic/untreated] and group II [NOD diabetic MK0431/treated].	After characterization of the diabetic condition, animals of both groups presented glucose levels higher than 300 mg/dL.23 Then, the animals of group II received MK0431 mixed in a pelleted diet (11 g/kg). group I received pelleted diet and water ad libitum, however, without hypoglycaemic agents. Group II was treated for 4 weeks with Sitagliptin. Glucose levels were monitored, and salivary gland samples were collected for histological analysis of extracellular matrix fibrillar components by polarized light microscopy.	Body weight variation was not significantly different [*P* > 0.05]. Significant reduction of the glucose levels was observed in animals of group II treated with Sitagliptin [*P* < 0.05]. the salivary glands of diabetic animals were target of this hyperglycaemic condition, presenting structural changes, characterized by pleomorphic acini, minor spatial area occupied by secretory epithelium and a higher volume density of collagen fibres. In contrast, recovery of the glandular structure was observed in the group treated with MK0431. The submandibular glands of treated animals presented a higher recovery, characterized by a minor quantity of collagen fibres and organization of secretory epithelial cells.
11.	Pradeep et al. 2013 [54]	India	In-vivo	Explore the efficacy of 0.5%, 1%, and 1.5% MF gel as a local drug delivery system in adjunct to SRP for treatment of intrabony defects in patients with chronic periodontitis.	MF	Placebo or 3 test groups: 0.5% MF, 1% MF, and 1.5% MF	Clinical parameters like modified sulcus bleeding index, plaque index, probing depth [PD], and clinical attachment level [CAL]. Data were recorded at baseline, 3, and 6 months, and radiographic and intrabony defects were recorded at baseline and 6 months. Mean concentration of MF in GCF was estimated by liquid chromatography.	Mean PD reduction and mean CAL gain were found to be greater in MF groups than in the placebo group at both 3 and 6 months, and greater reduction of intrabony defects was found in the MF groups compared to the placebo group. Local delivery of MF increases the PD reduction, CAL gain, and improves the depth reduction.
12.	Hashiguchi et al., 2014. [47]	Japan	Animal study	Assessed the effect of Volgibose on implant integration using diabetic rats.	Voglibose	10 Goto-Kakizaki (GK) rats were given an oral antidiabetic agent at a dose of 1 mg/kg in their drinking water [case group]. The remaining animals acted as a control and received no drug [diabetic group]. As an additional control, 5 male Wistar rats were maintained on a standard diet [CE-2; CLEA] and received no therapeutic agents [Control group].	Titanium screws were surgically implanted in each tibia of 20 GK rats and 5 non-diabetic control rats. After 3 or 9 weeks, osseointegration was determined by testing the removal torque required to displace the screw and by histological analysis of various parameters of bone formation.	Removal torque was significantly higher in the nondiabetic control group than in GK rats, irrespective of whether the GK rats had received voglibose. Histology revealed that the single-labeled surface area was still high in the GK rats at 9 weeks but had peaked and diminished in control rats. Bone-implant contact area was reduced in GK rats. Despite controlling blood glucose, voglibose was unable to reverse the bone metabolic effects of DM.
13.	Inouye et al., 2014 [15]	United States of America	Animal Study	To assess the effects of hyperglycemia and MF administration on periimplant healing	MF	Thirty-six rats were divided into three groups: non-diabetic rats [controls], GK spontaneously diabetic rats; GK rats were fed MF [100 mg/kg body weight per day] for 4 weeks.	The right maxillary first molars region, where Titanium implants were placed, was used. Six rats from each group were analyzed at weeks 1 and 4 by micro-computed tomography for bone/implant contact ratio, percent bone volume, trabecular number, and bone mineral density. Blood was also analyzed for glucose, HbA1c, and pyridinoline [PYD].	At week 1, glucose levels in the GK-Met rats were high, and all bone parameters were similar to those of GK rats [lower bone parameters and higher PYD than controls]. At week 4, glucose levels in the GK-MF rats and all parameters were similar to those of controls. Hyperglycemic GK type 2 diabetic rats showed improved blood glucose and wound healing around oral implants after MF administration.
14.	Wu et al., 2014 [57]	China	In-vivo	Potential bone-sparing effect of insulin in diabetic Periodontal disease	Insulin	Three groups: Control: normal culture conditions [5.5 mM]; with 25 mM and with 45 mM glucose	The hPDL cells were obtained from healthy hPDL tissue and were treated with high concentrations [25 or 45 mM] of glucose with or without different concentrations [10^−6^, 10^−7,^ or 10^−8^ mM] of insulin.	High concentrations of glucose increased the production of IL-1β, TNF-α, and IL-6 at both mRNA and protein levels, and RANKL at mRNA levels in hPDL cells. It has a stimulatory effect on pro-inflammatory cytokines and RANKL expression and upregulates OPG expression. Insulin exerts its bone-sparing effects on periodontal tissues by altering the inflammatory cytokines in hPDL cells.
15.	Pradeep et al., 2015 [55]	India	In-vivo	Evaluated the efficacy of open-flap debridement [OFD] combined with Platelet-rich fibrin [PRF], 1% MF gel, and PRF + 1% MF gel in the treatment of intra-bony defects in patients with chronic periodontitis.	MF	4 groups: OFD alone; OFD with PRF; OFD with 1% MF; OFD with PRF plus 1% MF	% reduction in depth of bone defect, site-specific plaque index, modified sulcus bleeding index, probing depth [PD], relative attachment level [RAL], and gingival marginal level were recorded before surgery and 9 months postoperatively	Groups with PRF and MF showed significantly more PD reduction and RAL gain than the OFD alone group. Mean PD reduction and mean RAL gain at 9 months were found to be greater in the group with 1% MF and PRF compared to the group with ODF 1% MF. The PRF + 1% MF group showed greater improvements in clinical parameters, with a greater percentage of radiographic defect depth reduction.
16.	Bastos et al., 2017 [19]	Brazil	Animal	To evaluate the effects of MF on bone healing around titanium implants inserted in non-diabetic rats.	MF	20 Wistar rats were divided into: control group [*n* = 10]: rats without MF treatment; MF group [*n* = 10]: rats treated with MF [40 mg/kg/day].	30 days after implant placement, histometric measurements of bone area and bone-to-implant contact, in addition to immunohistochemical analysis of the number of cells stained for RANKL and OPG, were assessed in the cortical and medullary areas around implants.	The percentages of bone area and bone-to-implant contact in the cortical bone were significantly lower in the MF group than in the control group [*P* < 0.05]. Furthermore, the medullary bone around the implants inserted in the MF group showed an increased number of RANKL-stained cells compared to that around the implants inserted in the control animals [*P* < 0.05]. MF negatively affected osseointegration by reducing the percentages of bone area and bone-to-implant contact and increasing the expression of RANKL around titanium implants inserted in non-diabetic rats.
17.	Serrao et al., 2017 [14]	Brazil	Animal	To assess the effect of MF on the bone healing around implants in diabetic rats	MF	30 rats were divided into three groups: Type 2 DM without MF [*N* = 10]; Type 2 DM with MF [40 mg/kg/day] 15th day after implant placement [*N* = 10]; control group: non-diabetic rats without MF.	The titanium implants were placed in the tibiae at the end of the 30^th^ day, and 30 days after implant surgery, the following parameters were checked: Bone-to-implant contact, bone area, and the number of receptor activator of nuclear factor κB ligand [RANKL]- and osteoprotegerin [OPG]-stained cells were assessed in cortical and medullary areas.	The percentages of bone to implant contact and Bone area in the cortical bone decreased in the DM and DM on MF groups compared to the control group [*P* < 0.05]. The bone area percentage in the medullary region was lower in the DM group compared to the control group [*P* < 0.05]. The DM group with MF exhibited the highest number of OPG-stained cells, while the DM group had the highest RANKL/OPG ratio in the medullary area [*P* < 0.05]. Although MF did not mitigate the damaging effect of hyperglycemia on bone healing around implants at the histometric level, it increased OPG expression and decreased the RANKL/OPG ratio in the medullary area.
18.	Sun et al., 2017 [39]	China	Animal Study	Evaluated the effects of MF on orthodontic tooth movement in a rat model of type 2 DM	MF	Two groups: Type 2 diabetes group (*n* = 10) and an MF group (*n* = 10). The MF was given intragastric administration of metformin (100 mg/kg body weight) in phosphate-buffered saline every day for 1 month. The Type 2 diabetes group was given PBS only. Ten rats fed with standard rodent diet were injected with PBS intraperitoneally and given intragastric administration of PBS as a control group (*n* = 10).	Rats were fed a high-fat diet for 4 weeks to induce fat accumulation and insulin resistance, then injected with a low dose of streptozotocin (35 mg/kg) intraperitoneally to induce type 2 diabetes. An orthodontic appliance was placed in rats that were normoglycemic, had type 2 diabetes, or had type 2 diabetes with MF administration. Briefly, tooth movement was achieved using a coil spring delivering a force of 0.5 N (0.012-inch Ni–Ti wire; anchored to the maxillary right first molar and incisors, resulting in mesial tipping of the first molar.	After 14 days, type 2 diabetic rats exhibited greater orthodontic tooth movement and had more tartrate-resistant acid phosphatase-positive osteoclasts, stronger cathepsin K expression, and weaker alkaline phosphatase immunostaining than normoglycemic rats. MF administration resulted in normalization of osteoclast numbers, cathepsin K immunostaining, and tooth movement, as well as partial recovery of alkaline phosphatase expression in diabetic rats. MF also reduced sclerostin expression and improved the immunolocalization of dentin matrix protein 1 in osteocytes of type 2 diabetes rats. MF administration reversed the adverse effects of diabetes on orthodontic tooth movement.
19.	Houshmand et al., 2018 [22]	Iran	In-vitro	Effect of MF on the attachment of human dental pulp stem cells [hDPSC]	MF	4 groups: hDPSCs + macro-porous biphasic calcium phosphate [MBCP] + MF; hDPSCs + MBCP; hDPSCs + MF; discs alone [control] [*N* = 6]	Attachment of hDPSCs to bone granules in hDPSCs + MBCP + MF group and hDPSCs + MBCP was observed by scanning electron microscopy on days 1 and 7 of cultivation. Cell viability was assessed by MTT assay on days 1, 3, and 7 after cell seeding. Differentiation of the hDPSCs was assessed by measurement of alkaline phosphatase activity on days 3, 7, 14, and 21 after cell culturing in standard and osteogenic media.	MTT values increased in all groups from day 1 to day 7 [*p* < 0.001], with highest value in group with hDPSCs + MF followed by the control group and groups with tricalcium phosphate and hDPSCs [*p* < 0.001]. Alkaline phosphatase activity also increased in all groups between days 3 to 21 [*p* < 0.001] except between days 7 and 14 in standard media [*p* = 0.094]. In osteogenic media, the groups with MF showed higher alkaline phosphatase activity [*p* < 0.05]. MF increased the attachment and proliferation of hDPSCs on biphasic granules.
20.	Pankaj et al., 2018 [56]	India	In-vivo	Explore and compare the clinical efficacy of locally delivered 1.2% Rosuvastatin and 1% MF gel as an adjunct to scaling and root planning [SRP] in the treatment of intrabony defects in chronic periodontitis	RSV and MF	3 groups: SRP plus placebo gel; SRP plus 1.2% Rosuvastatin gel; SRP plus 1% MF gel.	Clinical parameters like modified sulcus bleeding index [mSBI], plaque index [PI], pocket probing depth [PD] and clinical attachment level [CAL] were recorded at baseline, 6, and 12 months, and the radiologic assessment of bone defect fill was performed at 6 and 12 months.	mSBI, BP, PD, and CAL were improved in all the groups, but mean reductions in PD, CAL gain, and percentage of bone fill were found to be higher in groups 2 and 3 than in group 1 at all visits. 1.2% Rosuvastatin and 1% MF gel caused a PD reduction, CAL gains, and improved bone fill when compared with placebo gel. The use of 1.2% Rosuvastatin gel was better than 1% MF gel.
21.	Qin et al., 2018 [27]	China	Invitro	Effects of MF on the proliferation and differentiation of DPCs.	MF	Dental pulp tissues were obtained from explants of clinically healthy dental pulp from human adult third molars that were removed from individuals undergoing tooth extraction for orthodontic treatment. Different concentrations of metformin were added, and the cells were incubated for the indicated time periods. In the experiments involving the AMPK inhibitor Compound C (EMD Chemicals, San. Diego, CA), Compound C was added 1 hour before the addition of metformin.	Cell proliferation was analyzed using a cell counting kit, and levels of phosphorylated and unphosphorylated AMPK were quantified by Western blot analysis. Effects of AMPK inhibitor Compound C were determined by alkaline phosphatase activity assay and von Kossa staining, and odontoblastic markers were evaluated by reverse-transcription PCR analysis.	The western blot assay showed that DPCs express functional organic cation transporter-1. MF significantly activated the AMPK pathway, stimulated alkaline phosphatase activity, enhanced mineralized nodule formation, and increased the expression of odontoblastic markers. AMPK inhibitor Compound C markedly reversed MF-induced odontoblastic differentiation and cell mineralization. MF induces DPC differentiation and mineralization in an AMPK-dependent manner.
22.	De Araújo et al., 2019 [42]	Brazil	Animal study	Effects of gliclazide on levels of oxidative stress, inflammatory markers, and bone loss	Gliclazide	Five groups [*n* = 10 each]: no ligature; ligature wire alone; ligature wire with 1 mg/kg gliclazide; 5 mg/kg gliclazide; 10 mg/kg gliclazide	Maxilla scanned using micro-CT to quantify linear and bone volume/tissue volume [BV/TV] and volumetric bone loss. Histopathological, immunohistochemical, and immunofluorescence analyses were conducted to examine levels of inflammatory markers [MMP-2], [COX-2]. RTPCR to quantify the gene expression of the nuclear factor kappa B p50 subunit [NF-κB p50], phosphoinositide 3-kinase [PI3k], protein kinase B [AKT]	1 mg/kg gliclazide reduced myeloperoxidase activity, malondialdehyde, IL-1β, and TNF-α levels [*p* ≤ 0.05], and resulted in weak staining for COX-2, Cathepsin K, MMP-2, RANK, RANKL, superoxide dismutase type 1 [SOD1], Macrophage migration inhibitory [MIF], and phosphoinositide 3-kinase [PI3K]. Treatment decreased neutrophil and macrophage migration, decreased the inflammatory response, and bone loss in rats with ligature-induced periodontitis.
23.	Bautista et al., 2019 [48]	Brazil	Animal study	Effects of sitagliptin on bone tissue and on implant osseointegration in diabetic rats.	Sitagliptin	4 groups [*n* = 32]: Diabetic animals [GD]; Diabetic animals sitagliptin; Normoglycemic animals; Normoglycemic animals that received sitagliptin.	Four weeks after streptozocin injection and confirmation of DM [with and without sitagliptin], implants were placed in the right tibiae. Sitagliptin or water was administered for 4 weeks. Glycemia, HOMA-IR [Homeostatic Model Assessment for Insulin Resistance], insulinemia, microtomographic parameters of the left tibia, and implant-bone area fraction occupancy [BAFO] was evaluated.	HOMA-IR results showed no insulin resistance, and insulinemia was lower in diabetic animals. Sitagliptin did not influence glycemic control. The diabetic animals showed a lower BAFO, bone volume fraction, and lower trabecular number and thickness. Sitagliptin has no direct action on bone tissue and has no protective bone action in decompensated diabetic animals.
24.	Kim et al., 2019 [49]	Korea	Animal study	Therapeutic effect of MF in non-obese diabetic mice.	MF	Eleven-week-old mice were orally administered 50 mg/kg MF daily for 9 weeks. [aged 7–9 weeks, female]	The mice were anesthetized and injected intraperitoneally with pilocarpine [5 mg/kg]. Saliva was collected from the oral cavity for 7 min in a microtube. The microtube containing the saliva was briefly centrifuged, and the volume was measured using a micropipette. Salivary flow rate was expressed in microliters per minute per gram of body weight [μL/min/g]. The salivary flow rate was measured at 11, 13, 15, 17, and 20 weeks. Histological analysis was done. CD4^+^ T and B cell differentiation and Serum total IgG, IgG1, and IgG2a levels were determined.	MF reduced salivary gland inflammation, restored the salivary flow rate and reduced the IL-6, TNF-α, IL-17 mRNA, and protein levels in the salivary glands, Th17 and Th1 cell populations, but increased the regulatory T-cell population in the peripheral blood.
25.	Kawahara et al., 2020 [33]	Japan	Animal and in vitro study	Evaluated the possibility of targeting the NOD-like receptor protein 3 [NLRP3] inflammasome pathway by glyburide to suppress periodontal pathogen-induced inflammation	Glyburide	64, 7-wk-old male Lewis rats [Oriental Yeast] were used in this study [*n* = 8/group]. Glyburide in 20% ethanol [20 mg/kg weight of rats] or vehicle [mock] was orally administered to each group every 24 h using a tube.	Human monocyte cell lines were differentiated to macrophage-like cells and stimulated with *Porphyromonas gingivalis, Aggregatibacter actinomycetemcomitans* or *Fusobacterium nucleatum*, in the presence of glyburide. IL-1β and caspase-1 expression in the cells and culture supernatants were analyzed by Western blotting and enzyme-linked immunosorbent assay, and cell death was analyzed by lactate dehydrogenase assay. 64 7-wk-old male Lewis rats were used [*n* = 8/group]. *P. gingivalis, A. actinomycetemcomitans*, or *E. coli* [0.6 µg/3 µL of PBS] was injected into the palatal gingiva of the maxillary first molar every 24 h. Glyburide in 20% ethanol [20 mg/kg weight of rats] or vehicle [mock] was orally administered to each group every 24 h using a tube. Five groups of serial sections, each containing 10 subsections, were obtained from each specimen, each subjected to hematoxylin and eosin staining for histopathological observation. The number of infiltrated inflammatory cells in the connective tissue above the bone crest was counted using image analysis software [Image J]. The distance between the cement-enamel junction [CEJ] and the alveolar bone crest was measured to assess bone resorption. To identify osteoclasts, sections were stained with tartrate-resistant acid phosphatase. The number of TRAP-positive cells on the alveolar bone surface on the periodontal ligament side was counted under the light microscope, and IL-1β production was detected immunobiologically.	Glyburide treatment suppressed IL-1β expression in culture supernatants and enhanced intracellular IL-1β expression. Oral administration of glyburide significantly suppressed the infiltration of inflammatory cells and the number of osteoclasts in the alveolar bone compared with the control. In addition to glyburide, glimepiride was shown to suppress the release of IL-1β from THP-1 macrophage-like cells. Oral administration of glyburide significantly decreased the number of inflammatory cells in rats injected with *A. actinomycetemcomitans* or *E. coli*. Oral administration of glyburide significantly decreased the distance between the CEJ and the alveolar bone crest in rats injected with each bacterium. The immunostaining of IL-1β. Oral administration of glyburide decreased the IL-1β expression in the gingiva of rats injected with each bacterium.
26.	Nishikawa et al., 2020 [40]	Japan	Animal Study	Investigated the effects of insulin on periodontitis without any local treatments for periodontitis under type 1 DM using the ligature-induced experimental periodontitis model.	Insulin	Two groups: normal rats, diabetic rats, and diabetic rats with insulin treatment [*n* = 20 in each group]. Six‐week‐old Sprague–Dawley rats were weighed after the overnight fasting and STZ (60 mg/kg was injected for the induction of diabetes. Rats with plasma glucose concentrations of >15 mmol/L were selected as the diabetic rats at 1 week after the STZ‐injection. The experimental animal groups were as follows: normal rats, diabetic rats and diabetic rats with insulin treatment (n = 20 in each group).	Nylon thread was ligated around the unilateral maxillary second molar. The other side was untreated as the control. (b) Streptozotocin (STZ) was intraperitoneally injected to induce diabetes. Two weeks after STZ injection, experimental periodontitis was induced by ligation. Half of the diabetic rats received insulin treatment for 2 weeks.Insulin treatment was carried out by the insertion of insulin pellets. The blood flow in the gingival tissue was measured using a laser Doppler blood flow meter. For messenger ribonucleic acid (mRNA) and protein analyses, gingival tissues were obtained. For immunohistological and micro‐computed tomography (CT) analyses, maxillary bones with gingiva on both sides were taken. Gene expression of gingiva using Total RNA was checked using RT-PCR. Maxillae were scanned by micro‐CT.	Insulin directly suppressed lipopolysaccharide-induced inflammatory cytokine expressions in THP-1 cells. Insulin treatment significantly improved inflammatory cell infiltration and inflammatory cytokine gene expression, leading to suppression of alveolar bone loss, in the periodontitis of diabetic rats. Insulin also suppressed the periodontitis-increased nitric oxide synthase-positive cells in the periodontal tissue of the diabetic rats. Diabetic rats showed decreased gingival blood flow and an increased number of nitric oxide synthase-positive cells in the gingiva and alveolar bone loss compared with normal rats, all of which were ameliorated by insulin treatment.
27.	Zhou et al., 2020 [38]	China	Animal study	To investigate the underlying mechanism between diabetic periodontitis and NOD-like receptor [NLR] family pyrin domain-containing 3 [NLRP3] inflammasome-associated pyroptosis	MF	3 groups: Thirty 5-week-old male BKS-Leprem2Cd479 db/db mice; Ten 5-week-old male C57BLKS were used as control. After a one-week quarantine, mice of 6 weeks of age were randomly divided into three groups: normal control [*N*], DM, DM and P.g. periodontal infection, or periodontal infection with MF groups of 10 mice each.	The NLRP3 inflammasome-related cytokines and gasdermin D [GSDMD] were assessed in vitro and in vivo. MicroCT analysis was used to examine the mandibular bone, ELISA was used to detect IL-1β levels in serum and immunochemistry analysis was used to detect GSDMD expression in gingival tissue. Small interfering RNA was used for transfection. Intracellular K⁺ was detected by inductively coupled plasma optical emission spectrometry. PI/Hoechst 33342 staining was performed, and the number of PI-positive cells was quantified. Western blot analysis was conducted on periodontal tissues from the lower jaw, which were prepared and tested for NLRP3, P2X7R, caspase-1, GSDMD, mTOR, ASC, and IL-1β. Quantitative RT-PCR [qRT-PCR] was performed to validate the mRNA of the differently expressed proteins NLRP3 and DGSDMD. Microarray analysis was performed to screen for differentially expressed long non-coding RNAs [lncRNAs].	Diabetes-associated periodontitis mice exhibited the worst fasting glucose and bone destruction. GSDMD-positive cells and NLRP3 inflammasome expression were augmented in the gingival tissue, which were partly reversed by MF. In vitro data show NLRP3 inflammasome stimuli induced cell pyroptotic death, and deletion of NLRP3 decreased GSDMD expression. MF ameliorates the outcome of pyroptotic death by inhibiting NEK7/ NLRP3 pathway.
28.	Zhao et al. (2020) [32]	China	In vitro	Assessed the osteogenic differentiation ability of Human exfoliated deciduous teeth (SHEDs).	MF	MF powder was dissolved in normal saline and then the solution filtered and sterilized with 0.22 μm microporous membrane to obtain 10 mM stock solution. The solution was diluted serially with a culturing medium to achieve the final concentrations of 0, 10, 50, 100, and 200 μM MF in the culture plates. After starving for 12 h on the culture medium containing 1% FBS, SHEDs were cocultured with the DMEM medium containing different concentrations of MF	SHEDs were isolated from the dental pulp of deciduous teeth from healthy children aged 6 to 12, and their surface antigen markers of stem cells were detected by flow cytometry. Real-time quantitative reverse transcription-polymerase chain reaction (qRT-PCR) was performed to detect the gene expression of runt-related transcription factor 2 (Runx2), type I collagen (COL-I), alkaline phosphatase (ALP), osteocalcin (OCN), bone morphogenetic protein 2 (BMP2), vascular endothelial growth factor A (VEGFA), receptor activator of nuclear factor-κB ligand (RANKL), and osteoprotegerin (OPG). The effect of MF (10–200 μM) treatment on SHEDs cell viability, proliferation, and osteogenic differentiation was analyzed. The activation of adenosine 5’-monophosphate-activated protein kinase (AMPK) phosphorylation Thr172 (p-AMPK) was determined by western blot assay.	MF (10–200 μM) did not affect the viability and proliferation of SHEDs but significantly increased the expression of osteogenic genes, alkaline phosphatase activity, matrix mineralization, and p-AMPK level expression in SHEDs. Compound C, a specific inhibitor of the AMPK pathway, abolished MF-induced osteogenic differentiation of SHEDs. Moreover, MF treatment enhanced the expression of proangiogenic/osteogenic growth factors BMP2 and VEGF but reduced the osteoclastogenic factor RANKL/OPG expression in SHEDs.
29.	Boreak et al., [2021] [26]	Saudi Arabia	In-vitro	Assess the influence of MF on the angiogenic ability of secretomes from dental pulp stem cells.	MF	4 groups: control [untreated]; 2.5 microM MF; 10 microM MF; 20 microM MF.	The stem cells were obtained from DPSCs using the explant culture method and treated with different concentrations of MF. The angiogenic effect of the secretomes on the yolk sac membrane of the chick embryos was assessed using the quaternary blood vessel formations.	No significant MF-pretreated SHEDs could be a potential source of seed cells during stem cell-based bone tissue engineering. Difference in the activity of DPSCs after MF treatment. A significant increase in the expression of the VEGFA, FGF2, and CXCL8 genes. Angiogenesis increased with increasing concentrations of MF. Pre-treatment with MF enhanced the angiogenic potential of the secretome of DPSCs.
30.	Gutkind et al. 2021 [60]	USA	In-vivo	Explore the potential of MF to target PI3K/mTOR signaling for head and neck squamous cell carcinoma [HNSCC] prevention.	MF	Twenty-two participants completed the 12–14 weeks of MF.	Participants received MF for 12 weeks [week 1, 500 mg; week 2, 1000 mg; weeks 3–12, 2000 mg daily]. Pretreatment and post-treatment biopsies, saliva, and blood were obtained for biomarker analysis [serum glucose, hemoglobin A1c [HbA1c], and C-peptide concentrations and serum and saliva MF concentrations	MF significantly reduced serum HbA1c levels [from 5.7% ± 0.5% to 5.5% ± 0.4%, *P* = 0.023], but did not affect glucose or C-peptide levels. The mean serum MF concentration was 705.0 ± 444.0 ng/mL following the intervention. MF could be detected in saliva, with an average concentration of 171.0 ± 143.3 ng/mL, which was significantly correlated with the serum concentration. The clinical response rate [defined as a ≥ 50% reduction in lesion size] was 17%. MF can be used as a chemopreventive agent.
31.	Kouhestani et al., 2021 [24]	Iran	In-vitro	The effect of MF on both the proliferation and osteogenic capability of dental pulp stem cells (DPSCs) cultured on FDBA granules.	MF	Cellular viability and the osteoinducing effect of 100 μmol/L MF on DPSC were confirmed followed by loading on DBA granules and treated with and without MF.	SEM was done to check for cell attachment; MTT assay to check for proliferation and alkaline phosphatase (ALP) activity analysis osteogenic differentiation.	The SEM images revealed that MF enhanced the adhesion of DPSCs on FDBA granules. In addition, metformin was shown to increase cell proliferation/viability from day 1 to day 7. Compared to the control, a significant difference was observed after 7 days of treatment. MF enhanced the osteogenic capability of FDBA in both standard and osteoinducing conditions. An increase in ALP activity was significant after 7 days of treatment. The positive effect of MF on differentiation was significant in osteoinducing conditions.
32.	Liu et al., 2021 [13]	China	In-vitro	To assess the effects of sitagliptin on *Porphyromonas gingivalis*-lipopolysaccharide [LPS]-induced inflammatory response in human gingival fibroblasts [HGFs]	Sitagliptin	HGFs were isolated, and the effects of LPS and sitagliptin (at 0.1, 0.25, and 0.5 μmol·L^−1^, 5-1 000 μmol·L^−1^) on cell viability were detected.	HGFs were isolated, and the effects of LPS and sitagliptin on cell viability were detected using a cell-counting kit [CCK-8]. The mRNA levels of interleukin [IL]-6, IL-8, C-C motif ligand 2 [CCL2] and superoxide dismutase 2 [SOD2] were evaluated using quantitative real-time polymerase chain reaction [qRT-PCR]. ELISA was then used to measure the protein secretion levels of IL-6, IL-8 and CCL2. Western blot analysis was then used to investigate the activation of the NF-κB signaling pathway further. The effect of the NF-κB pathway inhibitor BAY11-7082 on LPS-induced inflammatory cytokines in HGFs at the gene level was verified by qRT-PCR.	qRT-PCR analysis found that 0.5 µmol·L-1 of sitagliptin and 5 µmol·L-1 of BAY11-7082 significantly inhibited LPS-induced IL-6, IL-8, CCL2, and SOD2 gene expressions. Sitagliptin significantly inhibit LPS-induced HGF inflammatory response by blocking the NF-κB signaling pathway activation.
33.	Mohamed Abdelgawad et al., 2021 [31]	Egypt	In-vitro	Evaluated the effects of photobiomodulation therapy along with MF on the proliferation and viability of human periodontal ligament stem cells [HPDLSCs] that are cultured in high glucose medium.	MF	HPDLSCs were divided into eight groups: Group I [control], non-diabetic HPDLSCs without irradiation; group II, diabetic HPDLSCs without irradiation vitamin D alone, without irradiation; group III, diabetic HPDLSCs with laser irradiation at 1 J/cm2; group IV, diabetic HPDLSCs cultured with MF and laser irradiation at 1 J/cm2; group V, irradiation at 2 J/cm2; group VI: MF and laser irradiation at 2 J/cm2; group VII: cells received 3 J/cm^2^ laser irradiation and group VIII: HPDLSC with MF and laser-irradiated at 3 J/cm^2^. Addition of extra glucose to diabetic groups 24 h before cell irradiations	MF was added to half of the diabetic groups. The cells were irradiated with an 808 nm diode laser after 24 and 48 h. Cell viability was analysed using an MTT assay 24 h after irradiation to detect cell viability in each group. Real-time PCR was used to evaluate the gene expression of Nrf2, Keap1, PIK3 and HO-1, as well as the effect of PBMT on the Keap1/Nrf2/HO-1 pathway. An ELISA reader was used to evaluate cell viability through measuring ROS, TNF-α and IL-10 protein levels after cell irradiation.	Combining photobiomodulation at 3 J/cm² with MF enhanced the proliferation and viability of the HPDLSC diabetic cell lines, and improved their differentiation and function with minimal side effects. Photobiomodulation at 1, 2 and 3 J/cm² combined with MF significantly increased the viability of HPDLSCs. There was an increase in TNF-α, IL-10, and the gene expression of Nrf2, Keap1, PIK3, and HO-1 [*p* < 0.05].
34.	Shen et al., 2021 [18]	Japan	Animal study	Effects of exendin-4 on orthodontic tooth movement	Exendin-4	Exendin-4 solution [0.2 μg, 4 μg, 20 μg] or PBS	20 microliters of exendin-4 solution were injected on the buccal side of the upper left first molar at a 2-day interval. Mice were sacrificed on day 12; silicone impressions were taken to record tooth movement distance.	Orthodontic tooth movement distance was smaller in the 20 μg exendin-4 group than in the PBS group [*P *< 0.01]. Compared with the PBS group, the 20 μg exendin-4 group showed lower osteoclast number [*P *< 0.05] and odontoclast number [*P *< 0.05].
35.	Shi et al., [2021] [58]	China	In-vivo	Assess the radiographic marginal bone loss and clinical parameters around implants in patients using different hypoglycemic agents.	Insulin, MF, or glucagon-like peptide-1 [GLP-1] drugs	Three groups [patients included 150; implants included 308]: Insulin [*N* = 54]; MF [*N* = 54]; GLP-1 drugs [*N* = 42].	Patients received implant placement, and prosthesis restorations were ceramic crowns. The peri-implant marginal bone levels were evaluated by periapical radiographs immediately after implant placement at 1 and 2-year follow-up visits.	A total of 71 patients with 129 implants completed the two-year follow-up: 30 patients with 53 implants in the insulin group; 24 patients with 44 implants in the MF group; and 17 patients with 32 implants in the GLP-1 drug group. The GLP-1 drug group exhibited significantly less peri-implant marginal bone loss than the other two groups [*P* < 0.01]. At the one-year follow-up, mesial and distal marginal bone loss [MBL] in the GLP-1 drug group was smaller than in the MF and insulin groups. Similarly, the percentage of mesial marginal bone loss to implant length in the MF group [4.30% ± 1.58%] was significantly higher than in the GLP-1 drug group [3.67% ± 1.50%] [*P* < 0.01]. At the two-year follow-up, the mesial and distal MBL parameters in the insulin group were smaller than in the MF group [*P* < 0.05]. Regarding clinical parameters at the two-year follow-up, there was no significant difference in BOP [+] [*P* > 0.05] and the mean PD in the insulin group [1.30 ± 0.24 mm] was comparable to that in the MF group [1.37 ± 0.24 mm] and the GLP-1 drug group [1.27 ± 0.31 mm] [*P* > 0.05]. GLP-1 drugs have a positive effect on peri-implant bone remodeling.
36.	Xu et al., 2021 [37]	China	Animal study	Scaffolds loaded with MF [β-tricalcium phosphate [β-TCP], chitosan [CTS], and mesoporous silica [SBA-15].	MF	Three groups: Experimental group: alveolar bone defects implanted with MF/β-TCP/CTS/SBA-15 scaffolds; Control group: alveolar bone defects implanted with β-TCP/CTS/SBA-15 scaffolds; Blank group: no material was implanted.	MF-loaded β-TCP/chitosan/SBA-15 composite scaffolds were implanted into periodontal defects in rats with periodontitis. Scaffold properties were characterized using Scanning Electron Microscopy, X-ray Diffraction, and Fourier Transform Infrared Spectroscopy. Bone regeneration was assessed via micro-CT and histological analysis.	Bone marrow mesenchymal stem cell [BMSC] expression levels of the BMP-2, COL1a1 and Runx2 genes were higher in the MET/β-TCP/CTS/SBA-15 group than in the β-TCP/CTS/SBA-15 group. Most of the bone defects in the MET/β-TCP/CTS/SBA-15 group were repaired; however, the bone defect in the β-TCP/CTS/SBA-15 group remained, with limited repair, and there was almost no repair in the blank group. New bone formation occurred at the centre and periphery of the bone defect in the MET/β-TCP/CTS/SBA-15 group, whereas new bone formation was limited to the area around the junction of the β-TCP/CTS/SBA-15 scaffold and the bone defect. The MET/β-TCP/CTS/SBA-15 group exhibited abundant collagen fibre formation. Unlike normal bone tissue, little bone formation occurred in the central area of the defect and the arrangement of new bone was disordered in the β-TCP/CTS/SBA-15 group.
37.	Zhang et al. [2021] [23]	China	In-vivo	Inhibitory effect of MF on human Dental pulp stem cells [hDPSCs] senescence	MF	Two groups with 6 donors per group: young group [18–27 years] and aging group [65–74 years]	DPSC obtained from the crown and superior two-thirds of the root pulp were cultured with hDPSCs and treated with MF. The expression of microRNA-34a-3p was assessed using quantitative PCR.	100 μM MF promoted the proliferation of young DPSCs from days 1 to 3 [*p* < 0.05]. 250 μM MF inhibited the proliferation of ageing DPSCs on days 9 and 10 [*p* < 0.01]. At 500 μM, a decrease in proliferation activity was noted in young DPSCs on days 9 and 10, and in ageing DPSCs after day 5 [*p* < 0.05]. MF alleviated DPSC senescence by downregulating miR-34a-3p and upregulating CAB39 via the AMPK/mTOR signaling pathway.
38.	Zhang et al., 2021 [45]	China	Animal study	Investigated the effects of Genipin and insulin treatment on osseointegration of dental implants in T2DM rats	Genipin + Insulin	Rats with DM were divided into five groups [*N* = 6 in each group]: Healthy control group; Rats with Type 2 DM; DM rats treated with Insulin [10 IU/kg]; DM rats treated with Genipin [50 mg/kg]; DM rats treated with Genipin and insulin combination	Serum glucose levels and weight were checked for all rats. After three months, acid-etched [SLA] sand-blasted titanium implants were placed in the distal femurs of each rat. The femora were then harvested for computerised tomography analysis. The following parameters were assessed: volume fraction, mean trabecular number and percentage of osseointegration. Immunohistochemical staining was performed for phospho-AMPK and 8-OHdG [8-hydroxy-2′-deoxyguanosine]. Pull-out tests were performed for biochemical testing, and different histomorphometric assessments were conducted to evaluate bone-to-implant contact and the percentage length of the direct bone-to-implant interface in relation to the total implant surface in the cancellous bone.	Oxidative stress and blood glucose levels were higher in the untreated DM group, which was correlated with poor osseointegration. Combination treatment lowered glucose levels and reactivated the AMPK signaling pathway in the Type 2 DM group. This therapy also reduced oxidative stress and reversed the negative effects on osseointegration. While satisfactory healing was observed in the DM groups administered genipin or insulin alone, the combination of genipin and insulin was found to be more effective in promoting implant osseointegration.
39.	Lu et al. [2022] [53]	China	In-vitro	Synthesis of MF-based carbon nanodots [MCDs] and their effects on the biocompatibility and odontoblastic differentiation of hDPSCs	MF	Three groups: Control Group; MCD group; autophagy inhibition group.	MCDs were synthesized via hydrothermal treatment. MF-based MCDs were synthesized and characterized to investigate their effects in-vitro on odontoblastic hDPSC differentiation and the underlying mechanism. MCDs were synthesized by a hydrothermal treatment method and characterized using transmission electron microscopy (TEM), Fourier transform infrared spectroscopy, and X-ray photoelectron spectroscopy. hDPSCs were treated with MCDs [up to 200 μg/ml] to assess viability and differentiation. Odontoblastic differentiation was evaluated by expression of DSPP, DMP1, RUNX2, and SP7. Autophagy activation was confirmed as the key pathway using autophagy inhibitors.	MCDs at concentrations of 50, 100, and 200 μg/ml significantly promoted cell proliferation at 3, 7, and 10 days, while concentrations of 400 and 800 μg/ml MCDs inhibited cell proliferation at 10 and 14 days. MCDs promoted odontoblastic differentiation of hDPSCs by upregulating DSPP, DMP1, RUNX2, and SP7. MCDs activated autophagy and were biocompatible.
40.	Alshibani et al., 2023 [30]	Saudi Arabia	In-vitro	Effects of MF on the matrix metalloproteinases [MMPs] and proinflammatory cytokines production from lipopolysaccharide [LPS] stimulated human gingival fibroblasts [HGFs].	MF	1-control and 8 Experimental samples (0.5, 1 and 2 mM concentrations of MF).	HGFs were obtained from biopsies of healthy gingival tissues of patients undergoing oral surgeries and then incubated with MF and *Porphyromonas gingivalis* LPS. MMP-1, MMP-2, MMP-8, MMP-9, IL-1β, and IL-8 expression analysis was performed using xMAP technology.	Concentrations of 0.5, 1, and 2-m of MF had a minimal non-significant cytotoxic effect on the HGFs and caused a statistically significant reduction of MMP-1, MMP-2, MMP-8, and IL-8 expressed by the LPS-stimulated HGFs. MF has a minimal cytotoxic effect on gingival fibroblasts and causes a statistically significant reduction in MMP-1, MMP-2, MMP-8, and IL-8.
41.	Guo et al. [2023] [44]	China	Animal study	Role of glipizide in the pathophysiology of periodontitis.	Glipizide	Group-4 [*N* = 8 in each group]: Control group; Periodontitis group; periodontitis with 5 mg/kg glipizide group; Periodontitis with 10 mg/kg glipizide group.	The treatment of ligature-induced periodontitis in mice with different concentrations of glipizide involves assessing the levels of periodontal inflammation, alveolar bone resorption, and osteoclast differentiation. Inflammatory cell infiltration and angiogenesis are analysed using RT-qPCR, immunohistochemistry and ELISA.	Glipizide reduces alveolar bone resorption and the degradation of periodontal tissue, as well as the number of osteoclasts in periodontal tissue affected by periodontitis. In mice, reduced microvessel density and leukocyte/macrophage infiltration were also observed. Glipizide inhibits angiogenesis, the inflammatory phenotype of macrophages, and osteoclastogenesis, thereby alleviating the pathogenicity of periodontitis.
42.	Hong et al., 2023 [36]	Taiwan	Animal study	Effects of MF on osteoblast differentiation and osteoclast formation in cultured cells and its ability to heal the apical periodontitis rat model.	MF	Murine pre-osteoblasts MC3T3-E1 and macrophages RAW264.7 were incubated, and Cells were cultured under hypoxia [2% oxygen] or normoxia [21% oxygen] to assess the effect of MF. Ten 7- to 8-week-old rats were used to induce apical periodontitis in bilateral mandibular first molars.	Murine pre-osteoblast cells were treated with 50 or 100 ng/mL of soluble RANKL. MF was added to the cultures at concentrations ranging from 50 to 200 mM, three h prior to the other treatments. The pre-osteoblasts and macrophages were cultured under hypoxic conditions and then stimulated with RANKL after the addition of MF. Immunohistochemistry was performed to examine Runt-related transcription factor 2 [RUNX2], RANKL and the osteoclast marker tartrate-resistant acid phosphatase. For the animal study, apical periodontitis was induced in the lower first molars of rats. Root canal treatment was performed with or without MF supplementation. The pulp chambers were left open to the oral cavity for four weeks to allow the condition to develop. MF paste [1%] in propylene glycol was applied to the distal canal of the right mandibular molar. Micro-computed tomography was performed four weeks after root canal therapy to assess the extent of the lesion.	MF reversed hypoxia-induced RUNX2 suppression and RANKL synthesis in pre-osteoblasts. It inhibited hypoxia and RANKL-enhanced TRAP synthesis in macrophages. Intracanal MF diminished bone loss in rat apical periodontitis. MF is an effective medicament for inflammatory bone diseases.
43.	Sobhnamayan et al. [2023] [53]	Iran	In-vivo	Assessed the effect of MF in double antibiotic paste [DAP] on the regeneration process of non-vital immature teeth.	MF	Two groups: DAP group [*n* = 15] and DAP + MF group [*n* = 11]	Clinical and radiographic examinations were performed to evaluate the resolution of apical periodontitis, root development, and the occurrence of intracanal calcification	MF could promote root development in the regeneration process when incorporated in DAP. The rate of apical closure and root length was significantly higher in group with MF [*P* = 0.047]. However, the results were significantly similar in terms of root width [*P* = 0.184]. Canal obliteration was seen in 15% of cases with DAP + MF.
44.	Mohsen et al. [41]	Egypt	Animal Study	To assess and compare the therapeutic effect of bone marrow mesenchymal stem cells (BM-MSCs) versus insulin on mandibular dento-alveolar complex collagen formation and β-catenin (β-catenin) expression in experimentally induced type I diabetes in albino rats.	Insulin	Twenty-eight male albino rats were equally divided as follows: Group I: was composed of rats that received no drug. The remaining rats were administered a single streptozotocin (STZ) (40 mg/kg) intraperitoneal injection.	After affirmation of diabetes induction, the rats were divided into: Group II: Diabetic rats were given no treatment. Group III: Diabetic rats received a single BM-MSC intravenous injection (1 × 10^6 cells). Group IV: Diabetic rats were given a daily subcutaneous insulin injection (5 IU/kg). After 28 days, mandibles were processed and stained by Hematoxylin & Eosin, Masson’s trichrome, and anti-β-catenin antibody.	Dento-alveolar complex tissues and cells of Group II showed destructive changes histologically, while Groups III and IV demonstrated improved histological features. Group II presented almost old collagen in all dento-alveolar complex tissues, and nearly negative β-catenin expression. Groups III and IV revealed a newly formed collagen intermingled with very few areas of old collagen, and both groups showed positive β-catenin immunoreactivity. Statistically, Groups III and IV represented the highest mean values of Masson’s trichrome area% and β-catenin area%, while Group II reported the lowest mean. Streptozotocin has a destructive effect on the dento-alveolar complex structure and function. BM-MSCs and insulin show regenerative capacity in STZ-affected periodontal tissues, and statistically, they increase collagen formation and β-catenin expression.
45.	Jung et al. [2024] [50]	Korea	Animal study	Effects of gemigliptin on salivary gland hypofunction in diabetic rats.	Gemigliptin [GG]	Four groups were examined: normal rats [normal, *n* = 8]; diabetic rats [DM, *n* = 8]; DM rats treated with gemigliptin 10 mg/kg BW [DM-Ge10, *n* = 8]; DM rats treated with gemigliptin 100 mg/kg BW [DM-Ge100, *n* = 8].	GG was orally administered daily for 3 weeks. Anesthetized rats were given pilocarpine hydrochloride [2 mg/kg, IP], and saliva was collected for 15 min using pre-weighed cotton balls.	GG increased salivary flow rate and amylase levels, while reducing oxidative stress markers and alleviating histopathological damage to the salivary glands. These effects were associated with a reduction in the accumulation of advanced glycation end products [AGEs] and reactive oxygen species [ROS], as well as an increase in aquaporin 5 expression. GG is an effective treatment for patients with diabetes-related salivary gland dysfunction.
46.	Lingling et al., 2024 [28]	China	In-vitro	Effects and underlying mechanisms of insulin on the bone formation capability of human DPSCs.	Insulin	Three group: 0 M insulin + 0 µM LY294002 [control group]; 10^−6^ M insulin, 10 µM LY294002; 10^−6^ M insulin + 10 µM	Cell proliferation was assessed using a Cell Counting Kit-8. The cell phenotype was determined using flow cytometry. Colony-forming unit-fibroblast ability and multilineage differentiation potential were assessed using Toluidine blue, Oil Red O, Alizarin Red and Alcian Blue staining. Gene expression was examined using PCR, and protein expression using Western blotting. Bone regeneration was assessed using microCT, fluorescent labeling, immunohistochemistry and haematoxylin and eosin staining.	Insulin promotes the proliferation, osteogenic differentiation and bone formation capability of human DPSCs, and induces insulin resistance in them. 10^–6^ M insulin upregulates the expression of genes and proteins related to osteogenic differentiation that increase bone secretion metabolism. Insulin inhibits the expression of the insulin/insulin-like growth factor-1 signaling [IIS] pathway and the phosphorylation of PI3K and PI3K/total PI3K. Implanting insulin into the jawbone defects of mice and rabbits resulted in enhanced bone formation.

*IGF* insulin growth factor, *PCR* polymerase chain reaction, *DPSCs* Dental pulp stem cells, *MF* Metformin, *RANKL, OPG* MC3T3-E1, *HGF* human growth factor, *IL* Interleukin, *LPS* lipopolysaccharide, *TNF* tumor necrosis factor, *COX* cyclooxygenase, *BIC* Bone to implant contact, *BA* bone area, *NoD* non-obese diabetic, *TZD* Thiazolidinediones, *AMPK* Adenosine 5’-monophosphate-activated protein kinase, *hPDL* human periodontal ligament, *DSPP* Dentin Sialophosphoprotein, *DMP1* Dentin Matrix Protein 1, *RUNX2* Runt-related Transcription Factor 2, *SP7* Sp7 Transcription Factor, *RT-PCR* Reverse transcription-polymerase chain reaction, *DM* diabetes, *ELISA* Enzyme-linked immunosorbent assay, *IGF* Insulin-derived growth factor, *hDPSC* Human dental pulp stem cells, *GK* Goto-Kakizaki.

## Results

### Characteristics of individual studies

A total of 3061 studies were screened, of which 177 were included for full-text screening. Upon full-text screening, 131 articles were excluded (supplementary Table 1). Hence, 46 articles were included for the final review (Tables 1 and 2). Among the articles included, 13 were in-vitro studies [21–34]; 25 were animal studies [14, 15, 18–21, 33, 35–52]; 9 were in-vivo/ human studies [23, 53–62]. There were 14 studies on Insulin [20, 21, 28, 34, 40, 41, 43, 45, 46, 52, 53, 57–59], 23 on MF [14, 15, 19, 22–24, 26, 27, 29–32, 35–39, 49, 53–55, 58, 60], one on Exedin [18], one on Glicazide [42], two on Sitglitin [48, 51], one on Volibose [47], one on Glyburide [33], one on Glipizide [44], one on Gemigliptin [50], Genipin and insulin combination [45], one on MF and rosavastatin combination [56] and one on GLP-1 [45]. Based on the country-wise distribution, three studies were from India [54–56], five from Japan [18, 21, 33, 40, 47], 14 from China [20, 23, 27, 28, 32, 34, 37–39, 44, 45, 53, 57, 58], two from the United States of America [15, 50], Three from Korea [45, 55, 58]; Six from Brazil [14, 19, 33, 47, 57, 63]; two from Egypt [35, 36], one from Argentina [64], one from Saudi Arabia [24], one from Taiwan [56], one from the United Kingdom [59], two from Iran [22, 65] [Table 2].

### Critical appraisal and results from the individual sources of evidence

Anti-diabetic medications have both positive and negative effects on the gingival and periodontal soft tissues, alveolar bone, cementum, teeth, salivary glands, dental pulp, and oral mucosa. The overview of the effects of various anti-diabetic medications have been discussed as follows:

#### a. Effect on teeth

Anti-diabetic medications like MF (1,1-dimethylbiguanide hydrochloride), insulin, and exendin have been shown to have osteogenic effects and stimulate dental mesenchymal stem cells by inducing the differentiation and mineralization of pre-osteoblasts into osteoblasts via activation of the AMP-activated kinase (AMPK) signaling pathway. MF increases odontoblastic differentiation of dental pulp stem cells (DPSCs) and increases mineral synthesis in dentin and alveolar bone. MF also increases the vascularization potential of hDPSCs by inducing a marked increase in angiogenic factors like angiogenin and Vascular Endothelial Growth Factor (VEGF) from the stem cells [18–20, 66]. Insulin affects the induction of amelogenin and ameloblastin proteins, which are associated with the repair and regeneration of lost periodontal and pulpal tissues. Insulin also interacts with the insulin-like growth factors (IGF-I/ II) and affects the process of enamel biomineralization. Insulin regulates the transcription of ‘enamel-specific extracellular matrix proteins’ and aids in facilitating repair and osteogenic effects [21]. The dental pulp stem cells (DPSCs) are commonly used for the regeneration of pulpal tissues, as they have excellent odontogenic potential and should be explored further.

**Fig. 3:**
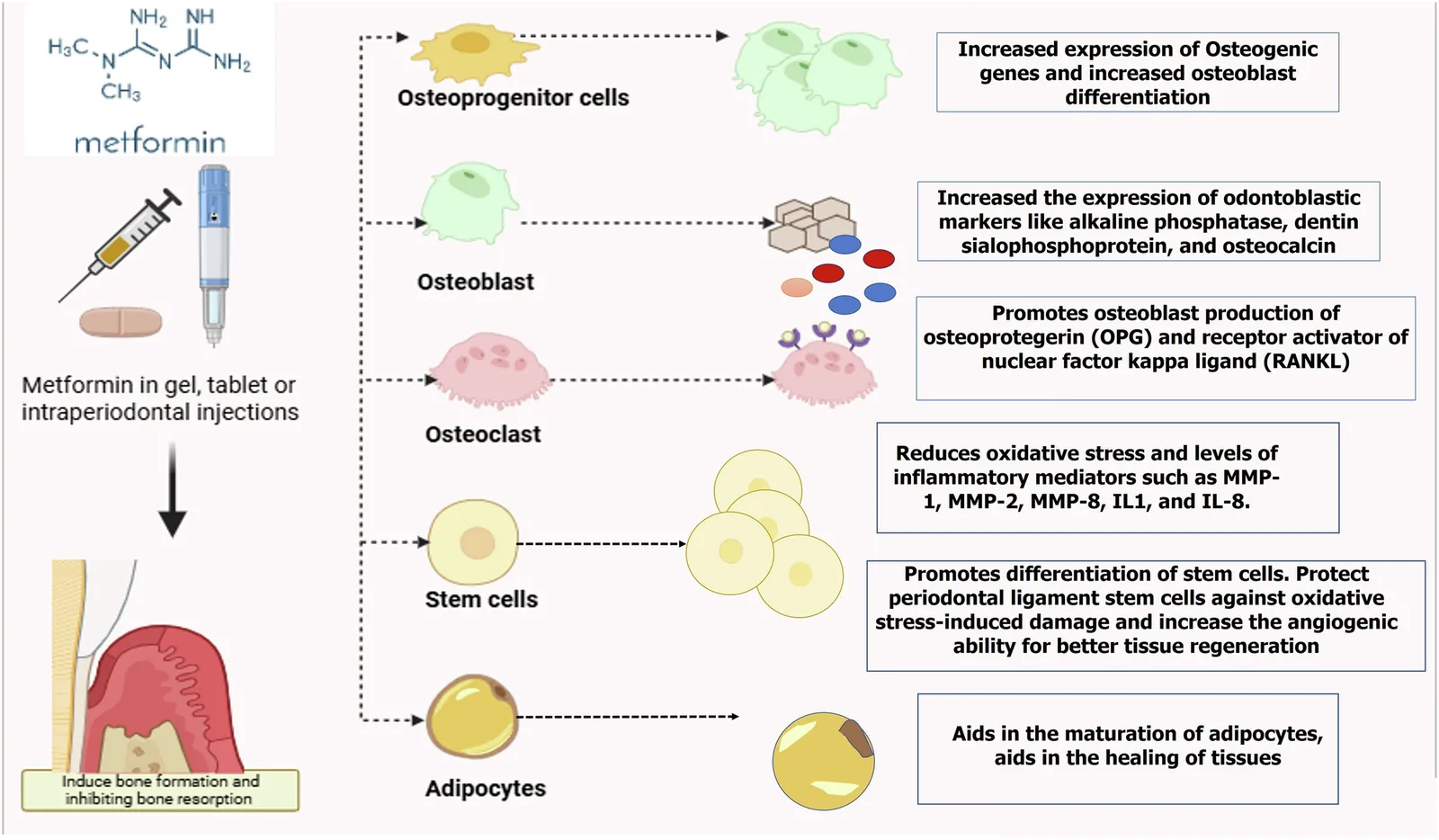
Effects of MF on oral tissues [Created in Biorender.com].

MF has also been shown to enhance periodontal regeneration by increasing the proliferation and attachment of hDPSCs by increasing the alkaline phosphatase activity [22]. A study by Zhang et al. (2021) found that MF increases the proliferation of DPSCs. MF inhibited senescence in DPSCs and significantly suppressed microRNA-34a-3p (miR-34a-3p) expression, elevated calcium-binding protein 39 (CAB39) expression, and activated the 5’ adenosine monophosphate-activated protein kinase (AMPK)/mammalian target of rapamycin (mTOR) signaling pathway. Furthermore, the authors found that transfection of miR-34a-3p mimics promoted the senescence of DPSCs, while MF treatment or Lenti-CAB39 transfection inhibited cellular senescence. These results indicated that MF could alleviate the senescence of DPSCs by downregulating miR-34a-3p and upregulating CAB39 through the AMPK/mTOR signaling pathway [65]. Sobhnamayan et al. (2023) investigated the combined effects of incorporating MF into the double antibiotic paste containing metronidazole and ciprofloxacin as an intracanal medicament during endodontic procedures in children for regenerating the non-vital immature teeth. After 18 months, a good rate of closure and an increase in root length were noted in patients where double antibiotic paste mixed with MF was used [*P * =  0.047]; however, the two groups were not significantly different in terms of root width (*P* = 0.184). Canal obliteration was seen in 15% of cases (*n* = 4), who were all among those treated with double antibiotic paste per se (Fig. 3) [53].

MF has also been mixed with bone graft to increase bone regeneration. A study by Houshmand et al. (2018) found that MF can increase the attachment and proliferation of human DPSCs (hDPSCs) and increase the alkaline phosphatase activity from the stem cells when cultured with a biphasic calcium phosphate graft. The cell viability increased from day 1 to day 7 (*p * <  0.001) upon application of MF. The highest cell vitality was noted (*p * <  0.001). The activity of alkaline phosphatase was also found to increase linearly from the third day to the 21st day after using MF [22]. MF has also been mixed with DPSCs cultured on FDBA granules for enhancing the proliferation and osteogenic capability of the graft. Kouhestani et al. (2021) conducted a pilot study on DPSCs to confirm cellular viability and the osteoinducing effect of 100 μmol/L MF. The SEM images revealed that MF enhanced the adhesion of DPSCs on FDBA granules. In addition, MF was shown to increase cell proliferation/viability from day 1 to day 7. An increase in ALP activity was significant after 7 days of treatment [24]. MF has also been incorporated into carbon nanodots to induce odontoblastic hDPSC differentiation. A study assessed the effect of MF-loaded carbon nanodots on hDPSCs and found that MF-loaded carbon nanodots were biologically safe, maintained cell viability, and induced apoptosis when used at 200 μg/ml. The MF-loaded carbon nanodots facilitated differentiation of hDPSCs by promoting differentiation of odontogenic cells via increased expression of odontoblast gene markers (RUNX2, DSPP, DMP1, and SP7) and proteins (DMP1 and DSPP). MF-loaded carbon nanodots also promoted odontoblastic hDPSC differentiation via auto cell death or autophagy [24]. MF also increases the vascularization potential of hDPSCs by inducing a marked increase in angiogenic factors like angiogenin and Vascular Endothelial Growth Factor (VEGF) from the stem cells. Boreak et al. assessed the influence of different concentrations of MF on the expression of the angiogenesis-related genes and angiogenic ability of hDPSCs. The study noted that MF increased the stem cells’ secretome angiogenic potential, and this helped to increase the blood vessel formations and expression of the VEGF, fibroblast growth factor (FGF), and CXCL8 genes. MF synergistically improved the mesenchymal stem cells / epithelial cells proliferation and promoted vascular formation [26]. MF promotes the regeneration of hDPSCs by activating the AMPK pathway in a dose-dependent manner. Qin et al. (2018) quantified the levels of ‘5′-monophosphate-activated protein kinase (AMPK), an important signaling pathway for regeneration. The author found that in response to MF, hDPSCs started expressing a transmembrane protein (functional organic cation transporter 1) that helped to mediate the intracellular uptake of MF. MF also increased the levels of alkaline phosphatase along with an increase in the odontoblastic markers, expression like dentin matrix protein 1, dentin sialophosphoprotein, osteocalcin, and runt-related transcription factor 2 [27]. MF can even inhibit hDPSCs’ senescence by downregulating ‘microRNA-34a-3p (miR-34a-3p)’ and upregulating the ‘calcium-binding protein-39 via activation of the mTOR signaling pathway. These properties of MF can be used to alleviate cellular senescence in DPSCs and confirm its regeneration potential. Insulin has also been shown to affect the bone formation capability of human DPSCs [Fig. 2].

Lingling et al. (2024) also found that insulin has regenerative potential as it can increase the proliferation of hDPSC, upregulate the genes linked with the differentiation of bone cells, and promote the formation of a mineralized matrix of bone. Insulin was also able to increase the secretion of proteins linked to bone metabolism and mineralized matrix formation. However, a significant inhibition of the expression of the insulin/insulin-like growth factor-1 signaling pathway was noted. An increase in the expression of the phosphatidylinositol-3-kinase (PI3K), phosphorylated PI3K/protein kinase B (AKT), total AKT, and mTOR was seen, proving the bone-forming properties of insulin [28]. Apart from insulin and MF, Exendin-4 has been shown to induce regenerative potential. Exendin is a peptide agonist of the glucagon-like peptide (GLP) receptors. It decreases the expression of Tumor necrosis factor-alpha (TNF-α) and, in turn, is able to reduce the resorption of roots in individuals with DM. Exendin intake reduced the number of osteoclasts, odontoclasts, and root resorption surface and rate of root resorption. Exendin-4 injections also decrease the mRNA expression for the ‘Receptor Activator of Nuclear Factor Kappa-B Ligand (RANKL), TNF-α, and RANKL/osteoprotegerin (OPG) production (*P* < 0.01). The inhibition of RANKL is the key mechanism by which Exendin-4 could inhibit orthodontic tooth movement. Thus, for DM patients undergoing orthodontic treatment and taking Exedin, additional precautions are needed while performing orthodontic treatment [27, 28].

#### b. Effect of anti-diabetic medication on the gingiva and periodontal tissues, including alveolar bone

Anti-diabetic medications such as insulin and MF have been used in gel, intraperitoneal, and intramuscular injections to reduce the inflammation of the periodontal tissues, improve the healing and regenerative capacity of the bone [54, 67, 68]. Anti-diabetic medication has been used as adjunct to are being used along with SRP for treating periodontitis and periodontal bone defects [54, 67–69]. Studies have proven that MF, along with SRP, significantly reduced pocket depth and clinical attachment loss, and aided in the regeneration of alveolar bone [69]. Local application of MF gel to the periodontal pockets or bone defects, along with SRP and flap surgery, has been shown to improve the regeneration of alveolar bone. The rationale of using MF for regeneration of bone is attributed to its ability to induce osteoblast activity, promote osteoblast production of osteoprotegerin (OPG) and receptor activator of nuclear factor kappa B ligand (RANKL), thereby inducing bone formation and inhibiting bone resorption [29, 54, 55, 64, 69–72]. MF can also increase osteogenic gene expression, induce osteoblast differentiation, and promote the maturation of adipocyte cells, which, in turn, facilitate the healing of alveolar bone [69, 70]. Liu et al. (2012) MF inhibits the periapical lesions possibly by lowering the RANKL/OPG ratio, subsequently reducing the number of osteoclasts and bone resorption areas. MF injections decreased the number of RANKL-positive and tartrate-resistant acid phosphatase (TRAP)-positive cells on day 14, whereas the number of OPG-positive cells increased on day 28. The periapical bone loss area in the MF-treated group significantly decreased on day 28 compared with the control group [72]. MF can augment the mineralization of immature osteoblasts derived from precursor cells. Various animal and in vivo studies have found that MF application helps in the regeneration of oral soft tissues and alveolar bone. For example, a study by Pradeep et al. tested the efficacy of use of 1% MF gel along with SRP and flap surgery resulted in more reduction in pocket depth, gingiva inflammation and bone loss in patients with periodontitis than the group that received SRP and flap surgery alone, comparing the use of MF with and without SRP showed that the application of MF with SRP gives better results and improvement in clinical probing depth. The application of MF, along with open flap debridement (OFD) or SRP, resulted in a significant improvement in interdental bone levels. He also reported that 1% MF is better than 0.5% MF and 1.5% MF for reducing periodontal inflammation [29, 72]. 1% MF can also be used along with platelet concentrates such as platelet-rich fibrin (PRF) for greater reduction in bone loss. The percentage reduction in radiographic defect depth upon using MF was higher compared to the use of PRF or OFD alone. The mean reduction in clinical probing depth and relative attachment level was greater in the PRF + 1% MF group compared to the group where PRF or MF was used alone at 9 months. At nine months, the radiographic defect depth reduction in the PRF + 1% MF group was higher (52.65% ± 0.031%] compared to PRF (48% ± 0.029%), MF (48.69% ± 0.026%], and OFD alone group (9.14% ± 0.04%) [55]. The application of MF gel (1% concentration) in the periodontal intra-bony defects was able to improve the differentiation of osteoblast cells and promote the process of bone formation. A study has also shown that when 1.2% Rosuvastatin and 1% MF gel were applied in the periodontal pockets after SRP for the management of intra-bony defects, it causes a significant reduction in probing depth and clinical attachment level, along with a gain in bone level. However, the results were better with the use of 1.2% rosuvastatin gel compared to 1% MF gel [55]. A meta-analysis in 2020 assessed the effect of application of 1% MF gel as an adjunct to SRP on periodontal intra-bony defects and reported that 1% MF application in intra-bony defects improves the clinical and radiographic outcomes; however, the certainty of evidence is moderate as the number of articles included for the review was limited. The quantitative analysis showed a weighted mean difference of 1.17 mm [*P* < 0.00001) for depth of periodontal intra-bony defect; a weighted mean difference of 2.54 mm (*P* < 0.00001) for clinical attachment level; a weighted mean difference of 2.01 mm (*P* < 0.00001) for probing depth; 0.38 (*P* = 0.002) for bleeding on probing; and a weighted mean difference of 0.00 mm (95% CI = − 0.04 to 0.04 mm, *P* = 0.95) for plaque index [73]. An animal study in mice by Bak et al. in 2010 also reported positive effects of using MF on the bone marrow cells and osteoblasts, and reported that MF can increase the osteoblast activity and its differentiation by approximately twofold. However, formation of osteoclast upon induction with 1,25-dihydroxyvitamin D3, lipopolysaccharide, or PGE2 was unaffected. A similar animal study comparing the effects of systemic MF for the management of periodontitis in rats reported a significantly lower rate of alveolar bone loss in groups receiving MF therapy compared to the control. MF improved the wound healing potential and reduced the degree of bone loss by inhibiting the interactions of advanced glycation end products with their receptors [35]. MF can reduce bone resorption by promoting osteoblast differentiation and diminishing osteoclast formation [36].

MF has also been used as a scaffold loaded with bone graft (β-tricalcium phosphate), chitosan, and silica. This novel scaffold containing MF increased the adhesion of bone marrow mesenchymal stem cells and promoted the repair of the bone defects [30, 31, 35–37, 73, 74]. Along with bone regenerative potential, MF also improved the healing of the gingiva and enhanced the regenerative potential of gingival fibroblasts. Alshibani et al. (2023) assessed the effects of MF on the matrix metalloproteinases enzymes and proinflammatory cytokines upon stimulation with lipopolysaccharide (LPS) on gingival fibroblasts. The authors found that MF reduces oxidative stress and levels of inflammatory mediators MMP-1/2/8, IL-1, and IL-8. This confirmed that MF is a good anti-inflammatory agent and has the potential to treat periodontal diseases [30]. MF has also been used along with photo-biomodulation therapy (photodynamic therapy), which involves the use of a laser and dye for better regenerative potential. The use of MF along with photo-biomodulation improves the proliferation and viability of cells with better osteogenic differentiation [31]. Combining MF with photodynamic therapy helped to reduce the production of free radicals, decrease the overall oxidative stress, increase the rate of apoptosis, reduce the nitric oxide levels, and decrease the expression of matrix metalloproteinase-2 (MMP-2) and cyclooxygenase-2 (COX-2) in the periodontal tissues. This combination also inactivates the nuclear factor kappa B (NF-κB) signaling pathway and reduces bone resorption [5]. MF has also been shown to act on the NOD-like receptors (NLRs). NLRP3 inflammasome-associated pyroptosis, which is the key receptor to inhibit the pyroptotic cell death of cells in periodontal tissues and reduce the inflammation in patients with DM [38]. Gong et al. (2023) developed a flexible MF-loaded silk fiber/gelatin patch with prolonged release of the drug to promote periodontal soft and hard tissue regeneration. The authors found that the MF-loaded silk patch was compatible with the oral tissues and showed pro-osteogenic, anti-inflammatory, and antioxidative properties. The patch also promoted M2 macrophage polarization and subsequent secretion of osteogenesis-related cytokines, which helped to achieve alveolar bone regeneration [63]. Sun et al. evaluated the effects of MF on orthodontic tooth movement in a rat model of type 2 DM. After 14 days, diabetic rats exhibited greater orthodontic tooth movement and had a higher number of tartrate-resistant acid phosphatase-positive osteoclasts, stronger cathepsin K expression, and weaker alkaline phosphatase immunostaining than normoglycemic rats. Metformin administration resulted in normalization of osteoclast numbers, cathepsin K immunostaining, tooth movement, and alkaline phosphatase expression in diabetic rats. MF also reduced sclerostin expression and improved the immunolocalization of dentin matrix protein 1 in osteocytes of type 2 diabetes rats [39]. Zhao et al. (2020) also found that MF significantly increased the expression of osteogenic genes, alkaline phosphatase activity, matrix mineralization, and p-AMPK level expression in SHEDs. Compound C, a specific inhibitor of the AMPK pathway, abolished MF-induced osteogenic differentiation of SHEDs. Moreover, MF treatment enhanced the expression of proangiogenic/osteogenic growth factors BMP2 and VEGF but reduced the osteoclastogenic factor RANKL/OPG expression in SHEDs [23].

Insulin has also been shown to have a bone-sparing effect as insulin signaling is essential for normal bone development and maintenance [31, 38, 39, 57, 63, 74]. Adequate insulin signaling helps protect, maintain, and promote the health of the alveolar bone and the tissues supporting the teeth, preventing premature bone loss and supporting regeneration. Wu et al. compared the effect of different concentrations of insulin on the periodontal tissues in the presence of hyperglycemia and found that insulin alters the expression of the inflammatory cytokines in PDL cells and maintains bone health [74]. Nishikawa et al. (2020) also found that insulin can improve the inflammation in periodontitis without any local treatment. Insulin administration lowered the infiltration of various inflammatory cells and reduced the expression of genes linked with the production of pro-inflammatory cytokines. This lowered the inflammation in periodontal tissues and alveolar bone. Insulin can even suppress the production of nitric oxide from monocytes, and this effect directly lowers the inflammatory cytokine expressions [41]. Mohsen et al. (2023) compared the effects of bone marrow mesenchymal stem cells (BM-MSCs) and insulin on collagen formation and β-catenin expression in experimentally induced type I diabetes in albino rats. The group of rats with no treatment showed destructive changes in connective tissue and the presence of old collagen in all dento-alveolar complex tissues, along with negative β-catenin expression. The groups treated with insulin and mesenchymal stem cells exhibited newly formed collagen intertwined with very few areas of old collagen, and both groups showed positive β-catenin immunoreactivity. In MSC-treated rats, the PDL appeared dense with fibers arranged in a regular pattern. Some cementoblasts and osteoblasts displayed a normal shape, while others appeared flattened or were absent in certain areas. Some cementocytes and osteocytes maintained a normal shape, whereas others were shrunken and altered. Most of the PDL fibrocytes exhibited a spindle shape, while others appeared flattened. In insulin-treated rats, both cementum and alveolar bone had a regular outline. The PDL was dense and regularly organized. Multiple cementoblasts and normal cementocytes were present. Oval osteoblasts, numerous osteocytes, and reversal lines were observed in the alveolar bone. Overlying PDL fibers, plump fibroblasts and flattened fibrocytes were noted. [41]

Apart from MF and insulin, other anti-diabetic medications like sitagliptin, gliclazide, and glimepiride have been shown to affect the regeneration of alveolar bone [39, 63]. Sulfonylureas such as glyburide, glipizide, and Gliclazide have also been studied for their role in reducing inflammation, bone loss, and vascular growth associated with the comorbidity of periodontitis and DM, as well as the molecular mechanisms involved [57]. A study by Araújo et al. (2019) found that gliclazide affects the levels of oxidative stress, inflammatory cytokines, and degree of bone loss in patients with periodontal disease. Gliclazide inhibits the PI3K signaling and decreases IL-1β and TNF-α levels in the periodontium. The reduction in these signaling pathways lowered the levels of ‘Matrix metalloproteinase-2 (MMP-2), RANK-RANKL, and cathepsin K enzymes. This provides a positive effect in reducing inflammation and alveolar bone resorption. Gliclazide has also been shown to inhibit bone loss by reducing the activation of the PI3K/AKT pathway, lowering neutrophil infiltration, and migration of macrophages [42–44, 75, 76]. Guo et al. [2023] assessed the effects of Glipizide on periodontal inflammation and alveolar bone and found that glipizide can reduce the loss of alveolar bone. It even reduced the infiltration of macrophages, activated PI3K/AKT signaling, reduced the formation of osteoclast cells, and promoted new blood vessel formation in periodontal tissue affected by periodontitis. However, no effects on the oral microbiota were noted [44].

Glyburide has also been tested as an anti-inflammatory agent to control periodontal inflammation. The glyburide assessed was able to target the “NOD-like receptor protein 3 (NLRP3)” inflammasome pathway and suppress the periodontal pathogen-induced inflammation. Glyburide was able to inhibit the release of IL-1β from the THP-1 human monocyte line [32]. Glyburide was also effective in inhibiting bone resorption in the periodontium following trauma. It was able to inhibit the NOD-like receptor protein 3/interleukin-1 beta pathway and activate caspase-1 enzymes, thereby reducing bone loss [77]. However, some anti-diabetic medications, such as thiazolidinediones, have been shown to reduce bone mineral density and increase osteoclastic activity, leading to bone resorption [78, 79]. Furthermore, Kawahara et al. (2020) found that glyburide could inhibit the release of mature IL-1β from THP-1 macrophage-like cells stimulated with periodontopathic bacteria (*Porphyromonas gingivalis, Aggregatibacter actinomycetemcomitans, or Fusobacterium nucleatum)*. THP-1 macrophages stimulated with periodontopathic bacteria secreted IL-1β without undergoing cell death. This process was inhibited by the NLRP3 inhibitor MCC950 and the caspase-1 inhibitor z-YVAD-FMK. Glyburide decreased IL-1β levels in the cell culture medium while increasing intracellular expression, suggesting that it may prevent the secretion of IL-1β. Furthermore, glyburide was found to decrease inflammatory cell infiltration, osteoclast formation, and bone resorption in rats with experimental periodontitis induced by periodontopathic bacteria [33]. Glyburide inhibits NLRP3 formation and induces the expression of pro-healing growth factors insulin-like growth factor-1 (IGF-1), transforming growth factor-β (TGF-β), and IL-10, thereby promoting wound healing in diabetic mice [45].

#### Effect on implants

Apart from treating periodontal bone defects, MF has also been used to treat peri-implant diseases. Various studies have reported mixed results regarding the use of anti-diabetic medication on the peri-implant soft tissue wound healing and osseointegration of bone around dental implants [45–49, 58, 80]. A review by Aljofi et al. (2023) found a positive/protective impact of MF on periodontium and peri-implant tissues and bone in DM patients. The use of MF caused a greater reduction in pocket depth, gain in clinical attachment level, and bone fill in intra-bony defects [77]. Inouye et al. (2014) investigated the impact of MF on the healing of peri-implant tissues. They examined the impact of MF therapy on bone-to-implant contact ratio, bone volume, number of trabeculae, and bone mineral density. Blood pyridinoline, HbA1c, and glucose levels were also examined. After one week, all healing and bone parameters were similar in diabetic rats. However, after four weeks, the glucose levels of the diabetic rats that received MF therapy were lower. The bone parameters were comparable to those of the healthy controls. These results suggest that diabetic rats exhibit improved glycemic control and enhanced healing around peri-implant tissues following MF treatment [15]. Morais et al. (2009) found that the bone formed alongside the thread was thinner in the saline group than in the insulin group. After four months, reduced bone density around the implants was noted in diabetic rats compared with insulin-treated rats. DM was found to impair bone density around the dental implant. However, insulin use could maintain bone density in DM rats [43]. However, a study by McCracken et al. reported that diabetic animals treated with insulin were not statistically different from those not treated. Although the Mean total bone volume percent (adjusted for day) for control, diabetic, and insulin groups (±SD) was 12.4 (±9), 22.6 (±10), and 17 (±7), respectively. Bone volume adjacent to implants in diabetic rats was significantly greater than controls (*P* < 0.05). Diabetic animals treated with insulin were not statistically different from controls [46].

In 2017, Wang et al. also evaluated the effect of locally applying controlled-release insulin to the implant-bone interface in Goto-Kakizaki (GK) Wistar rats with type 2 DM. Sprague-Dawley rats served as controls [*n* = 10]. The GK rats were divided into two groups: one with DM alone and one with DM and insulin application. Contact between the implant and bone, and the amounts of osteogenic and osteoid tissue and newly formed bone, were checked in both groups. Implant-to-bone contact was lower in the insulin group than in the control group. However, more new bone formation was observed in the insulin group. The authors concluded that, despite the degree of contact between the bone and implant being lower than in the control group, the infiltration of insulin during implant placement could improve implant-bone contact [20]. Additionally, the DM group exhibited the greatest bone loss. Shi et al. compared the effects of insulin, MF, and glucagon-like peptide-1 [GLP-1] on marginal bone loss around dental implants, noting that all of these anti-diabetic medications reduce marginal bone loss around implants. The peri-implant marginal bone loss in the GLP-1 group was significantly smaller than in the insulin and MF groups [*P* < 0.01]. MF produced a greater reduction in probing depth than insulin. The effects of MF on bone healing around titanium implants inserted in non-diabetic rats were evaluated.

Bastos et al. (2017) also investigated the impact of MF on osseointegration of dental implants in 20 Wistar rats, revealing that bone-to-implant contact and bone area in the cortical bone were notably lower in the MF group than in the control group (*P* < 0.05). The number of cells stained for RANKL and OPG in the cortical and medullary areas around the implants was higher than for the implants inserted in the control animals (*P* < 0.05). MF was found to negatively affect osseointegration by reducing the percentages of BIC and BA, as well as increasing the expression of RANKL, around titanium implants inserted in non-diabetic rats [19]. In a similar study, Serrao et al. investigated the impact of MF on bone healing around implants in diabetic rats. They discovered that MF did not offset the detrimental effects of hyperglycaemia on bone healing around implants at a histometric level. However, it increased OPG expression and reduced the RANKL/OPG ratio in the medullary area, offering potential molecular advantages for implant osseointegration under hyperglycaemic conditions [14]. The positive effects of glucagon-like peptide-1 (GLP-1) drugs on peri-implant bone remodeling were also noted, with results comparable to those of insulin or MF [46]. Voglibose, an oral α-D-glucosidase inhibitor used to manage postprandial hyperglycaemia in patients with DM, has been reported to be ineffective in improving bone regeneration. An animal study in rats using voglibose to improve osseointegration around implants reported no effect on the bone-to-implant interface. Furthermore, the removal torque was significantly higher in the non-diabetic control group than in the diabetic rat group taking Voglibose. Voglibose was unable to reverse the metabolic effects of DM on bone tissue [47, 58]. Bautista et al. (2019) assessed the effect of sitagliptin on bone tissue and implant osseointegration in diabetic rats, finding that it did not improve bone loss or the trabecular pattern. This confirms that sitagliptin does not reverse the harmful effects of DM on bone metabolism and osseointegration around implants [48, 49]. Zhang et al. (2021) tested the effects of combining Genipin and insulin to improve the osseointegration of dental implants in rats with type 2 DM. They found that this combination lowered blood glucose levels and activated AMPK signaling pathways. This lowered oxidative stress and reversed the negative effect on osseointegration. The combination of genipin and insulin was suggested as a promising method for enhancing the osseointegration of dental implants in diabetic conditions [45].

#### d. Effect on saliva

Anti-diabetic drugs like MF, Gemigliptin, Sitagliptin, and incretin have been shown to significantly influence the composition and function of the salivary glands. MF was found to be useful for reducing inflammation in the salivary gland and restoring the flow rate of saliva. Moreover, MF also decreased the levels of IL-6, IL-17, TNF, and protein levels in the salivary glands. An animal study on obese diabetic rats also found that MF was able to reduce the levels of Th1 and Th17 cells, along with an increase in the T-reg cells. MF was also found to suppress the effector T cells, induce regulatory T-cells, and regulate B cell differentiation. These mechanisms were useful for patients with xerostomia and Sjögren syndrome [45]. Nascimento et al. (2023) observed that MF enhances salivary gland secretion by inducing Ca²⁺ signaling, which restores saliva secretion and prevents immune cell infiltration in the salivary glands. MF-induced Ca²⁺ signaling inhibits the release of alarmins and prevents endoplasmic reticulum stress activation. This reduces immune cell infiltration and improves salivary gland dysfunction in patients with Sjögren syndrome [81]. The anti-fibrotic properties of MF are also useful for treating salivary fibrosis. Wang et al. found that MF can attenuate TGF-β1-induced fibrosis by inhibiting SMAD phosphorylation (*p* < 0.01) via AMPK-independent pathways, thereby activating the AMPK pathway and consequently suppressing NOX4 (*p* < 0.01), the main ROS producer. MF also protects acinar cells from ligation-induced injury, normalizing aquaporin 5 (AQP5) levels (*p* < 0.05) [46]. Gemigliptin has also demonstrated the ability to enhance salivary gland function and protect acinar cells [58]. It improves salivary gland function in diabetic patients by inhibiting apoptosis of salivary gland cells, reducing oxidative stress, improving salivary gland secretion, and increasing the expression of saliva-related proteins. Based on these results, gemigliptin has been proposed as an effective anti-diabetic drug for patients with hyposalivation [58]. Silva, Faria et al. [(013) also reported that anti-diabetic drugs such as dipeptidyl peptidase IV (DPP-IV) inhibitors can help to restore salivary tissue. This inhibitor helps maintain homeostasis and re-establish the epithelial and stromal compartments of salivary glands damaged by hyperglycaemia [51]. However, some studies have found that MF accumulates and is secreted in saliva. The presence of MF in saliva has been associated with taste disturbances, including a metallic taste in the mouth [82]. There is evidence that MF can partly restore the microbial flora in the saliva of diabetic patients. A study by Gu et al. (2011) found that MF use could restore the Acholeplasma and Comamonas genera in diabetic patients with periodontitis. Compared to patients with periodontitis, the genera Lactobacillus, Parvimonas, Norank_f_norank_o_Absconditabacteriales_SR1, and Acholeplasma changed significantly in diabetic patients taking MF compared to those not taking medication. The plaque index was positively correlated with Prevotella and Lactobacillus, but negatively correlated with Haemophilus, Lautropia, Unclassified_f_Pasteurellaceae, and TM7x. Treatment with MF was able to partially alleviate the alteration in salivary microbiota caused by Type 2 DM [83]. Insulin has also been shown to affect the stereology and morphology of the salivary gland. A study assessing the long-term effect of insulin on the morphology of the salivary glands in Nod mice found that alterations due to hyperglycemia, which are characterized by nuclear and cytoplasmic atrophy, biomembrane disorganization, an increase in fibrillar components of the extracellular matrix, and the presence of inflammatory cells, could be recovered by insulin. Insulin treatment exerted positive effects on the recovery of the changes resulting from the diabetic state in both parotid and submandibular glands [52].

#### f. Anti-diabetic medications and development of autoimmune conditions

Gliptins are associated with an increased risk of developing autoimmune diseases, including bullous pemphigoid, while being associated with mucous membrane pemphigoid. A case report by Hammami et al. (2015) on a 52-year-old man with Type 2 DM reported the development of severe mucosal erosions (Lichen planus) of the tongue, glans penis, and perianal area induced by glimepiride. Withdrawal of glimepiride, a Dipeptidyl peptidase 4 inhibitor (sitagliptin), has also been shown to cause oral ulcers [84]. Willis et al. (1999) also found that the presence of Candida species was present in all insulin-treated DM patients with and without clinical signs of infection. The study found that the development of oral candidiasis in insulin-treated DM patients is not the result of a single entity, but rather, a combination of risk factors [59]. Silvio et al. (2021) explored the potential of MF to target PI3K/mTOR signaling for HNSCC (head and neck squamous cell carcinoma) prevention and found that MF administration results in encouraging histological responses and mTOR (mammalian target of rapamycin) pathway modulation, thus supporting its further investigation as a chemopreventive agent [60].

## Summary of evidence and future research directions

Anti-diabetic medications have both positive and negative effects on the oral tissues. MF and insulin are two antidiabetic medications that have been shown to have osteogenic potential, as they can simulate dental mesenchymal stem cells by inducing the differentiation and mineralization of pre-osteoblasts into osteoblasts. The majority of the studies are done in animal models, with only a few clinical patient-based studies and in vivo studies. Studies have found osteogenic and mesenchymal stem cell proliferating effects of insulin and MF. MF activates the AMP-activated kinase (AMPK) signaling pathway and aids in odontoblast, osteoblast, and dental mesenchymal stem cells differentiation [24, 26–28]. The AMPK signaling pathway is involved in metformin-induced DPC odontoblastic differentiation and reparative process [27]. This facilitates osteoblast differentiation, promoting the differentiation of mesenchymal stem cells (MSCs) and pre-osteoblasts to osteoblasts to increase bone formation and increase the reparative process in periodontium. However, one should note that there is no direct evidence that MF causes regeneration of enamel tissue, and limited direct evidence or clinical studies exist to confirm the regeneration of enamel. While anti-diabetic medications such as metformin and insulin have been reported to influence cellular pathways involved in mineralized tissue formation, it is important to note that enamel regeneration does not occur in adults due to the absence of functional ameloblasts after tooth eruption. Therefore, any potential effects of these agents on enamel are limited to indirect or surface remineralization processes rather than true biological regeneration.” Furthermore, we would like to clarify that the primary regenerative potential of metformin, as demonstrated in recent literature, is related to its effects on dental mesenchymal stem cells, particularly dental pulp stem cells (DPSCs). Metformin has been shown to activate AMP-activated protein kinase (AMPK) signaling pathways, which enhance odontogenic differentiation, promote mineralization, and support reparative dentin formation. In addition, emerging evidence suggests that metformin can modulate the pulp microenvironment by reducing inflammation and enhancing angiogenic and regenerative responses, thereby contributing to pulp tissue repair and dentin–pulp complex regeneration. Recent studies have shown that MF promotes differentiation of DPSCs into odontoblast-like cells, increases expression of dentin-specific markers (DSPP, DMP-1), enhances mineralized matrix deposition, and supports reparative dentinogenesis in experimental models [23, 26–28, 31, 53, 73, 82].

MF and insulin have also been used in non-surgical and surgical periodontal therapy for the treatment of periodontal or peri-implant diseases. However, there is more literature on MF compared to other anti-diabetic medications. MF has been used as an adjunct to SRP and has shown promising effects for managing periodontal disease. MF and insulin have also been shown to induce bone formation in periodontal defects and facilitate bone fill. A meta-analysis assessing local 1% MF gel application noted that the certainty of evidence for improving clinical and radiographic outcomes in intra-bony defects is moderate due to the limited number of included articles [51, 52, 72, 82, 83]. Furthermore, comparative studies suggest that MF’s efficacy may be surpassed by other treatments; for instance, managing intra-bony defects showed better results with the use of 1.2% rosuvastatin gel compared to 1% MF gel [29–31, 35–38, 55, 56, 72–74]. Therefore, in addition to the recommended long-term comparative studies of MF versus other regenerative materials for periodontal and pulpal regeneration, new randomized controlled trials must focus on robust head-to-head comparisons against both non-anti-diabetic and emerging regenerative materials to solidify its precise clinical role. Clinical trials also needed to check the efficacy of osseointegration of dental implants, especially in patients with poor bone quality, those with osteoporotic bone, and diabetic patients [82, 83].

Future clinical and basic research must expand beyond MF and insulin to explore the effects of other anti-diabetic drugs on oral health, especially those demonstrating potent or adverse outcomes. GLP-1 drugs show significant promise in peri-implant applications, with one study noting that peri-implant marginal bone loss in the GLP-1 group was significantly smaller than in both the insulin and MF groups [20]. This suggests GLP-1 drugs should be a high priority for future peri-implant-bone remodeling research. Conversely, caution is warranted for specific drug classes, such as Thiazolidinediones, which have been shown to reduce bone mineral density and increase osteoclastic activity, thereby promoting bone resorption [32, 33, 43, 44, 75–79]. Long-term safety and epidemiological studies are also essential to track severe oral side effects, such as the increased risk of autoimmune conditions like bullous pemphigoid and mucous membrane pemphigoid associated with Gliptins, and mucosal erosions (Lichen planus) linked to glimepiride [50, 51, 81, 82, 85]. The dual nature of anti-diabetic medications, presenting both positive and negative effects on oral tissues [14–20], requires a precise definition regarding mechanism and application context. Specifically, research needs to clarify the conditional efficacy of MF. Mechanistic studies must be conducted to fully understand the specific signaling pathways and dose-dependent effects that modulate these regenerative responses, ensuring that the therapeutic application of MF is strictly guided by the patient’s underlying metabolic status.

## Conclusion

Anti-diabetic medications have a numeours  effects on the tissues and functions of the oral cavity. Various antidiabetic medications like insulin, MF, and other oral hypoglycemic agents have shown promising regenerative and reparative potential. They have been shown to stimulate the odontoblasts, the mesenchymal stem cells of the pulpal tissues, fibroblasts, and osteoblasts of alveolar bone to induce the reparative process and enhance healing. MF and insulin have shown to promote the process of bone formation and mineralization in pulpal, dentin, and alveolar bone in both in-vitro and in-vivo settings. However, evidence from human clinical studies is limited. Only few clinical studies explore the effect of these anti-diabetic medications, with most of the evidence evaluating the regenerative potential of MF and insulin as an adjunct to SRP for managing periodontal diseases and treating periodontal bone defects. Hence, long-term comparative studies on the efficacy of MF compared to other regenerative materials for periodontal and pulpal regeneration are needed. Additionally, future clinical and basic research that evaluate the long-term effects of these medication are needed. It also important to explore the effect of other anti-diabetic drugs on oral tissues, in periodontal regeneration and managing oral diseases, and for the maintenance of overall oral health.

## Supplementary information

List of excluded studies

## Data Availability

The data are available from the corresponding author upon reasonable request.
